# Filamentation and biofilm formation are regulated by the phase-separation capacity of network transcription factors in *Candida albicans*

**DOI:** 10.1371/journal.ppat.1011833

**Published:** 2023-12-13

**Authors:** Collin Ganser, Mae I. Staples, Maureen Dowell, Corey Frazer, Joseph Dainis, Shabnam Sircaik, Richard J. Bennett

**Affiliations:** Molecular Microbiology and Immunology Department, Brown University, Providence, Rhode Island, United States of America; University of Würzburg, GERMANY

## Abstract

The ability of the fungus *Candida albicans* to filament and form biofilms contributes to its burden as a leading cause of hospital-acquired infections. Biofilm development involves an interconnected transcriptional regulatory network (TRN) consisting of nine transcription factors (TFs) that bind both to their own regulatory regions and to those of the other network TFs. Here, we show that seven of the nine TFs in the *C*. *albicans* biofilm network contain prion-like domains (PrLDs) that have been linked to the ability to form phase-separated condensates. Construction of PrLD mutants in four biofilm TFs reveals that these domains are essential for filamentation and biofilm formation in *C*. *albicans*. Moreover, biofilm PrLDs promote the formation of phase-separated condensates in the nuclei of live cells, and PrLD mutations that abolish phase separation (such as the removal of aromatic residues) also prevent biofilm formation. Biofilm TF condensates can selectively recruit other TFs through PrLD-PrLD interactions and can co-recruit RNA polymerase II, implicating condensate formation in the assembly of active transcriptional complexes. Finally, we show that PrLD mutations that block the phase separation of biofilm TFs also prevent filamentation in an *in vivo* model of gastrointestinal colonization. Together, these studies associate transcriptional condensates with the regulation of filamentation and biofilm formation in *C*. *albicans*, and highlight how targeting of PrLD-PrLD interactions could prevent pathogenesis by this species.

## Author summary

The fungus *C*. *albicans* is a prevalent cause of life-threatening infections due, in large part, to its ability to form drug-recalcitrant biofilms on biotic or abiotic surfaces. Biofilm formation is controlled by a network of nine TFs that act in combination to regulate their own expression as well as that of downstream genes. We show that seven of the nine master biofilm TFs contain PrLDs, domains that have previously been shown to promote phase separation into a dense phase together with a more dilute surrounding phase. Importantly, disruption of the phase separation capacity of TFs by mutation of select PrLD residues blocks TF function both *in vitro* and during host colonization. PrLDs also promote the interaction of TFs with RNA polymerase II indicating that they can recruit key components of the cell’s transcriptional machinery. We therefore propose that TF PrLDs are integral to *C*. *albicans* biofilm formation due to their ability to promote protein-protein interactions and phase separation, and that the coordinated assembly of these complexes drives gene expression programs.

## Introduction

*Candida albicans* is an opportunistic human fungal pathogen found in 40–70% of healthy adults as a commensal organism [1]. This fungus can colonize multiple sites in or on the human body including the skin, mouth, gastrointestinal (GI) tract, and reproductive tract [2,3]. While often a harmless member of the human microbiome, shifts in pH levels, nutrition, disease state, or immune function can cause over-proliferation resulting in superficial mucosal infections or disseminated bloodstream infections [4,5]. Localized infections are also associated with implanted medical devices and the ability of *C*. *albicans* to form biofilms on either biotic or abiotic surfaces contributes significantly to disease progression in a clinical context [3,6].

*C*. *albicans* biofilms are multi-structured communities that exhibit increased resistance to antifungal drugs and physical disturbances compared to their planktonic counterparts [7]. This leaves limited treatment options for recurrent biofilm infections beyond the removal of medical implants or diseased tissues, or the use of higher drug doses which can have negative side effects for the patient [3]. Biofilms represent a central step in seeding new infection sites, and their continued presence in the host can lead to invasive candidiasis (IC)[2,8]. IC is the most common invasive fungal disease among hospitalized patients in the developed world with mortality rates close to 40% [4]. A better understanding of *C*. *albicans* biofilm formation is therefore essential for treating infections and managing chronic cases that are at risk of developing into IC.

Cells in *C*. *albicans* biofilms consist of three different morphologies–yeast, pseudohyphae, and true hyphae–which are encapsulated in an extracellular matrix (ECM) [3, 9]. Biofilm initiation involves adherence of yeast-form cells to a host surface or medical device [3]. Maturation is characterized by extensive filamentation and ECM development, with the latter consisting of proteins, carbohydrates, lipids, and nucleic acids [3,9]. During the dispersal phase of biofilm growth, yeast-form cells bud off from mature hyphal structures and can seed new infection sites [3,10].

Genetic screens have identified over fifty transcriptional regulators involved in *C*. *albicans* biofilm formation [3,11–13]. Deletion of any one of these regulators yields cells deficient in adherence, hyphal development or ECM production. In particular, nine transcription factors (TFs) are recognized as master regulators of biofilm development: Bcr1, Brg1, Efg1, Flo8, Gal4, Ndt80, Rob1, Rfx2 and Tec1 [11,12]. These TFs bind to the promoters upstream of their own genes as well as to the promoters of the other master regulators in a highly interconnected transcriptional regulatory network (TRN) [11,12]. Precisely how these proteins assemble at these regulatory regions to control biofilm formation is unknown and could involve both protein-DNA and protein-protein interactions.

Recent studies have postulated that TFs can control gene transcription through a phase separation mechanism [14–16]. Phase separation refers to the formation of two distinct phases from a single mixed phase, similar to oil droplets separating from water [17]. Membraneless organelles including the nucleolus, paraspeckles, and stress granules, are examples of structures formed via the phase separation of proteins and nucleic acids [18–21]. In a number of cases, phase separation has been linked to intrinsically disordered regions (IDRs) that can promote the formation of biomolecular condensates [17,22]. An important subset of IDRs includes low complexity sequences termed prion-like domains (PrLDs) that contain a high proportion of glycine and uncharged polar amino acids similar to classic yeast prions [23,24]. Several PrLD-containing proteins are prone to undergo liquid-liquid demixing and may also assemble into amyloid fibrils, including those that are associated with neurological diseases in humans [23–26]. Importantly, the deletion or substitution of specific amino acids within low complexity domains (LCDs) can restrict phase separation both *in vitro* and in the cell [25,27].

Recent work indicates that the TRN regulating the *C*. *albicans* white-opaque switch involves phase separation of PrLD-containing TFs [28]. The white-opaque switch is a bistable, heritable switch between two alternative cell states and, similar to biofilm formation, involves master TFs binding to large regulatory regions to regulate network output [29–33]. Deletion or mutation of the PrLDs within white-opaque TFs blocked their ability to phase separate and abolished their function in white-opaque switching [28]. Seven of the nine TFs in the biofilm network also contain PrLDs, including TFs that are shared with the white-opaque network, suggesting that these PrLDs may similarly play critical roles in biofilm formation.

In this study, we functionally analyzed the PrLDs present in multiple *C*. *albicans* biofilm TFs. We show that deletion of TF PrLDs, or substitution of key amino acids within these PrLDs, abolishes biofilm formation. The PrLDs present in these TFs undergo phase separation when expressed in the nuclei of mammalian cells, and mutations that block phase separation block their functionality in *C*. *albicans*. Two biofilm TFs, Efg1 and Flo8, phase separate to form liquid condensates that can incorporate the purified C-terminal domain (CTD) of RNA polymerase II, suggesting that condensate formation enables the assembly of active transcriptional complexes. Interestingly, PrLD swapping experiments revealed that the PrLD of mammalian TAF15 could substitute for that of Efg1 but not for that of Flo8, indicating that certain PrLDs are functionally interchangeable. We further show that the phase-separation capacity of TFs is necessary for their function in an animal model of colonization. Together, these findings reveal that TF PrLDs play key roles in regulating fungal filamentation and biofilm formation, and that disruption of the ability of PrLDs to phase separate can abolish pathogenic traits in *C*. *albicans*.

## Results

### Multiple *C*. *albicans* biofilm-regulating TFs contain PrLDs

**Fig 1:**
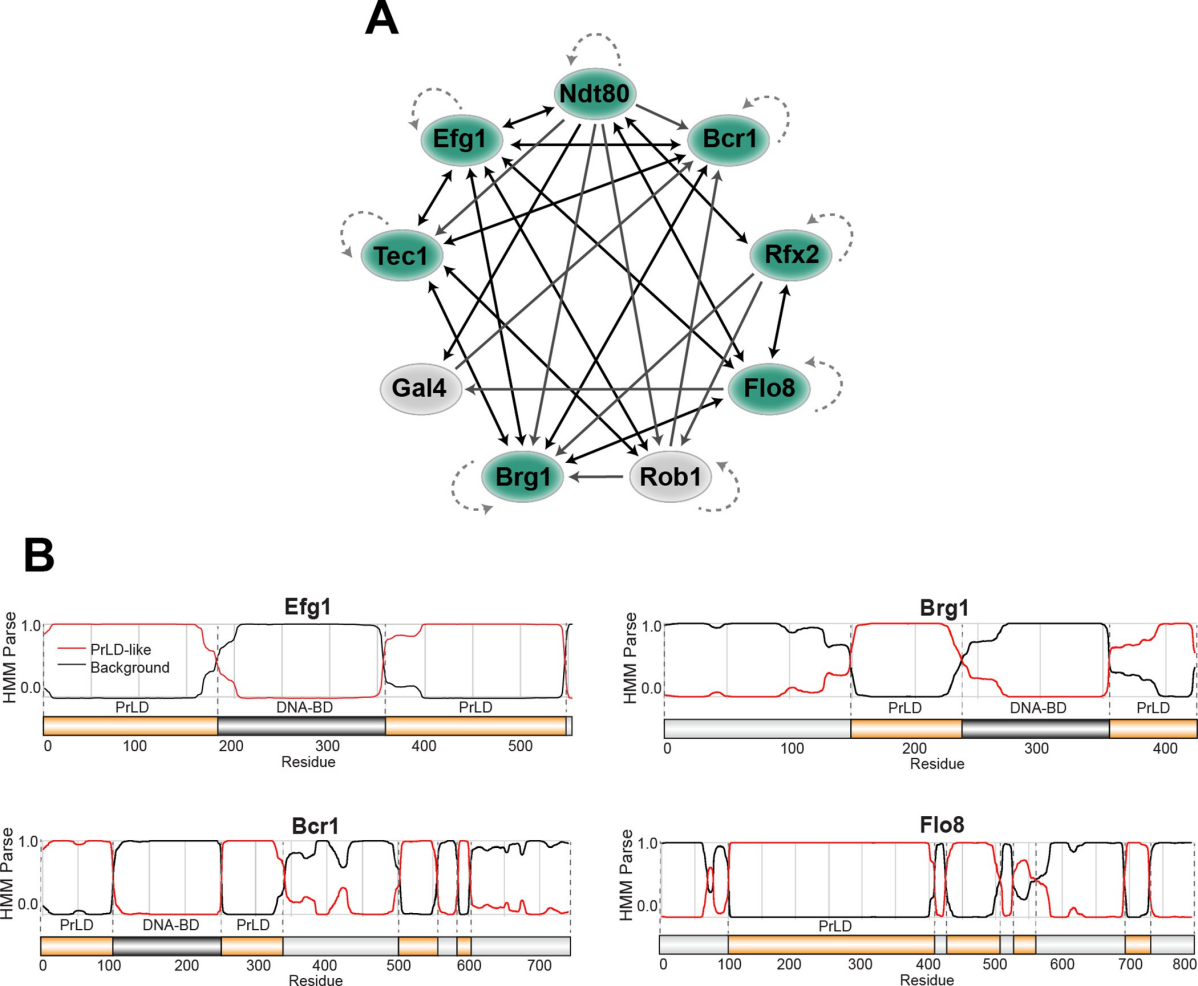
TRN controlling biofilm formation in *C*. *albicans* contains nine master TFs, seven of which contain PrLDs. **(A)** *C*. *albicans* biofilm TRN. Dotted arrows indicate autoregulation; solid arrows indicate binding of a protein to a given gene locus. TFs in green contain PrLDs; TFs in gray do not contain PrLDs. Adapted from Lohse *et al*. [3]. **(B)** PLAAC analysis of PrLDs. A hidden Markov model (HMM) defines protein regions as PrLDs or background [34]. Relative positions of PrLDs and DNA binding domains (DNA-BD) are shown.

The TRN controlling biofilm formation in *C*. *albicans* consists of nine master TFs that each autoregulate their own expression as well as that of the other genes in the network (**Fig 1A**) [11,12]. Chromatin immunoprecipitation assays have established that these master TFs bind at regulatory regions even in instances where consensus binding motifs are absent, suggesting that TFs are recruited to the DNA, at least in part, via protein-protein interactions [11,12]. Given that protein-protein interactions can be mediated by PrLDs that support the formation of complex biomolecular condensates [24,28], we evaluated biofilm TFs for the presence of these domains. Seven out of the nine biofilm TFs were found to contain substantial PrLDs, with Gal4 lacking any PrLDs and Rob1 containing only relatively short PrLDs (**Figs 1B** and **S1**). We chose four TFs based on their high PLAAC scores (prion-like amino acid composition scores) [34] to assess the role of PrLDs in the transcriptional regulation of fungal biofilm formation (**Fig 1B**).

### Functional analysis of TF PrLDs in biofilm formation

**Fig 2:**
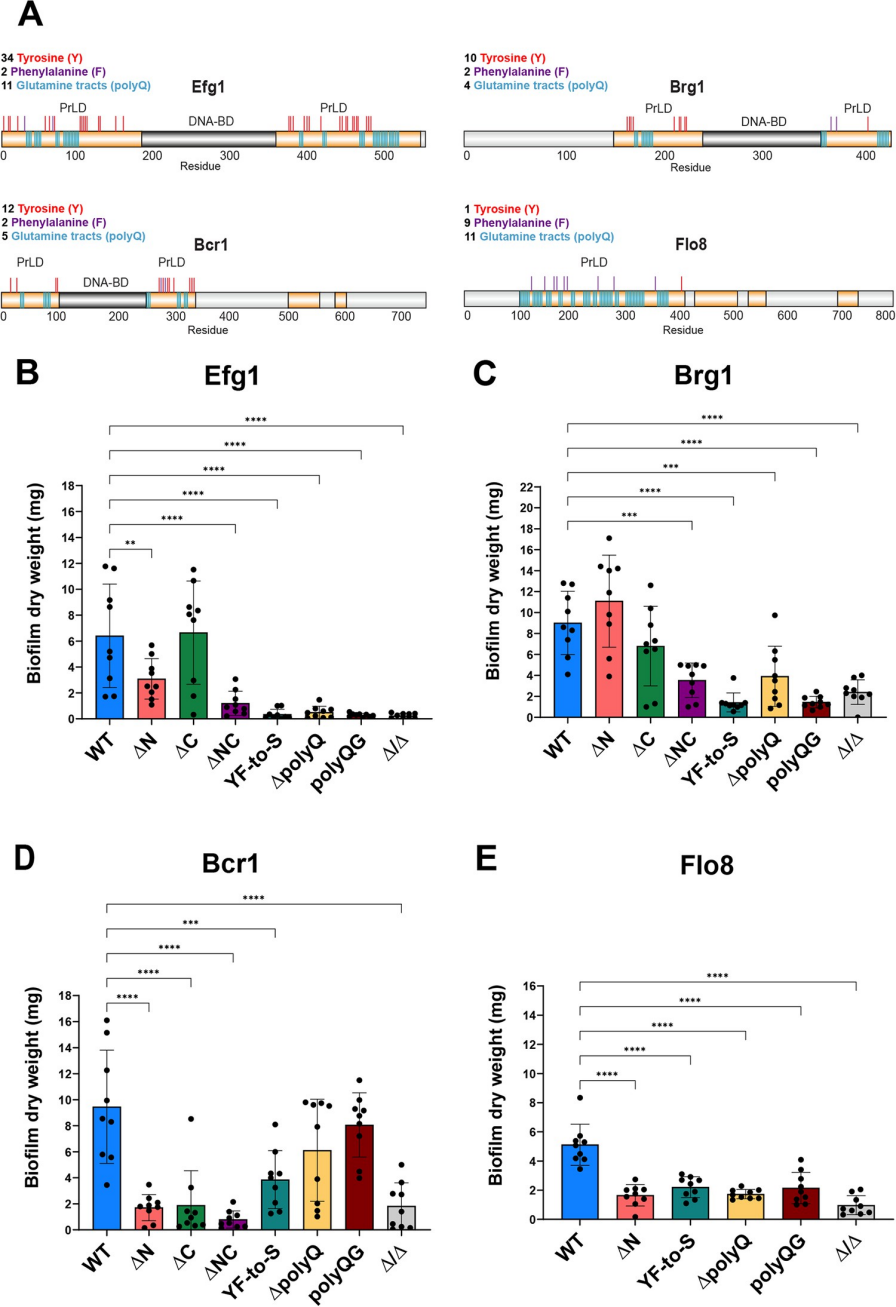
Dependency of biofilm formation on master TF PrLDs. **(A**) Schematic of specific amino acid residues in TF PrLDs. Relative positions and numbers of tyrosine (Y), phenylalanine (F), and glutamine tracts (polyQ) are shown. **(B-E)** WT or mutant strains (**B**, Efg1; **C**, Brg1; **D**, Bcr1; **E**, Flo8) were evaluated for biofilm mass on silicone squares. WT and mutants (ΔN, ΔC, etc.) refer to strains with one copy of the TF integrated into the corresponding TF Δ/Δ strain. Experiments were run in triplicate and each repeated three times. All statistical tests were performed using ordinary one-way ANOVA with Dunnett’s multiple comparisons test, in which the mean value for each mutant strain was compared to the mean value for the control. Error bars show S.D. Mutants without error bars did not have significant differences to the WT control. **P < 0.01; ***P < 0.001; ****P < 0.0001.

Three biofilm TFs (Bcr1, Brg1 and Efg1) contain two or more PrLDs, while one TF (Flo8) shows a high PLAAC score along most of its sequence with its largest single PrLD in the N-terminal region (amino acids 100–400; **Fig 1B**). We constructed TF derivatives that were lacking their largest one or two PrLDs or contained specific amino acid substitutions within these domains (**Fig 2A**). Substitutions targeted the aromatic amino acids tyrosine (Y) and phenylalanine (F) within PrLDs that can promote phase separation through pi-pi and cation-pi interactions [35–37], as well as stretches of glutamic acid residues (polyQ tracts) that can enable phase separation and/or aggregation [38–41]. PrLD mutants constructed included: (1) all Y and F residues changed to serine (YF-to-S derivatives); (2) deletion of polyQ tracts defined as three or more Qs in a row (ΔpolyQ); and (3) changing every other Q within polyQ tracts to glycine (polyQG). Wildtype or mutant TFs were integrated into *C*. *albicans* strains lacking the corresponding TF and examined for their ability to form biofilms on silicone squares (see **Materials and Methods**).

For each of the target TFs, loss of the PrLDs essentially abolished their ability to support biofilm formation. For example, in the case of Efg1, deletion of the N-terminal PrLD (ΔN mutant) or both the N- and C-terminal PrLDs (ΔNC mutant) prevented biofilm formation, whereas deletion of the C-terminal PrLD (ΔC mutant) did not significantly reduce biofilm mass compared to the full-length control (**Fig 2B**). The YF-to-S, ΔpolyQ and polyQG versions of Efg1 also showed a complete inability to form biofilms (**Fig 2B**). Brg1 similarly contains two PrLDs and loss of these domains resulted in an inability to form biofilms whereas deletion of either the N- or C-terminal PrLD did not significantly impact biofilm formation (**Fig 2C**). Brg1 YF-to-S, ΔpolyQ, and polyQG mutants were all highly defective in our assays (**Fig 2C**). Bcr1 also showed significantly decreased biofilm formation across all PrLD deletion mutants, including the ΔN and ΔC mutants, as well as the ΔNC mutant (**Fig 2D**). In contrast, however, while the Bcr1 YF-to-S mutant had decreased biofilm mass, biofilms formed by ΔpolyQ and polyQG mutants were not significantly decreased compared to the wildtype control (**Fig 2D**). Finally, deletion of the single PrLD in TF Flo8 resulted in loss of most biofilm formation compared to the full-length control (**Fig 2E**). Flo8 mutants also failed to form mature biofilms in any of the PrLD substitution mutants (YF-to-S, ΔpolyQ or polyQG; **Fig 2E**). We note that both Efg1 and Flo8 have a larger number of polyQ tracts (11 each) compared to Bcr1 and Brg1 (6 and 4 tracts, respectively), which could account for why deletion of these tracts has a larger impact in Efg1/Flo8 than in Bcr1/Brg1. Overall, these results reveal that PrLDs are critical for the function of master TFs in *C*. *albicans* biofilm formation, and that substitution of PrLD amino acids associated with phase separation capacities reduces or abolishes biofilm development.

**Fig 3:**
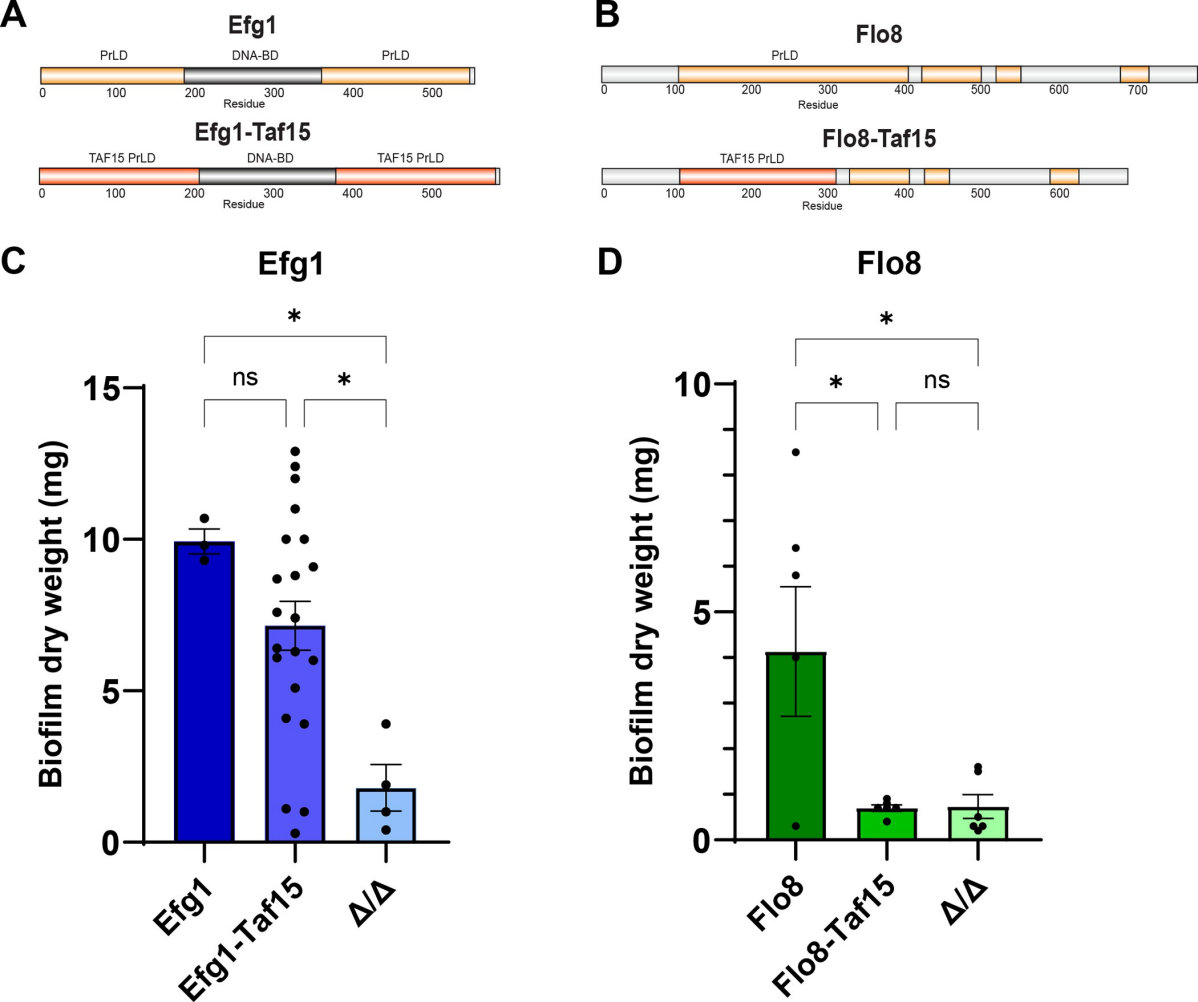
Analysis of biofilm formation supported by *C*. *albicans* TF-TAF15 PrLD chimeras. **(A,B)** Schematic of Efg1 and Flo8 sequences including chimeras constructed with the Taf15 PrLD. **(C,D)** Biofilm formation by Efg1- and Flo8-Taf15 chimeras. One copy of the WT or chimeric TF was integrated into the corresponding TF Δ/Δ strain. All statistical tests were performed using ordinary one-way ANOVA with Dunnett’s multiple comparisons test, in which the mean value for each mutant strain was compared to the mean value for the control. Error bars show S.E.M. *P < 0.05; ns = not significant.

We also created two TF chimeras to test if the PrLDs within biofilm TFs are interchangeable with an unrelated PrLD. To do this, the 204 residue PrLD from the human protein Taf15 was used to replace either the two PrLDs in Efg1 or the single PrLD in Flo8 (**Fig 3A and 3B)**. Interestingly, the Efg1-Taf15 chimera formed biofilms that were similar in mass to those of strains expressing WT Efg1 (**Fig 3C**), whereas the Flo8-Taf15 mutant was unable to form biofilms (**Fig 3D**). These results indicate that a PrLD from a human TF has the potential to functionally substitute for a fungal biofilm PrLD, but that not all PrLDs are equal and that such domain swapping is context dependent.

### Functional analysis of the master TF PrLDs in filamentation

Filamentation is closely associated with biofilm formation in *C*. *albicans*, as well as being integral to tissue invasion and virulence, and a number of biofilm-regulating TFs also control the filamentation program [3, 42, 43]. We therefore tested how mutations in biofilm TF PrLDs impact the yeast-to-hyphal transition. Cells were grown overnight in YPD (yeast peptone dextrose) medium at 30°C and then diluted into Spider medium, a strong inducer of filamentation and the medium used for biofilm assays. Cells were grown for 6 hours at 37°C in Spider medium and analyzed for yeast, hyphal, and pseudohyphal morphologies.

**Fig 4:**
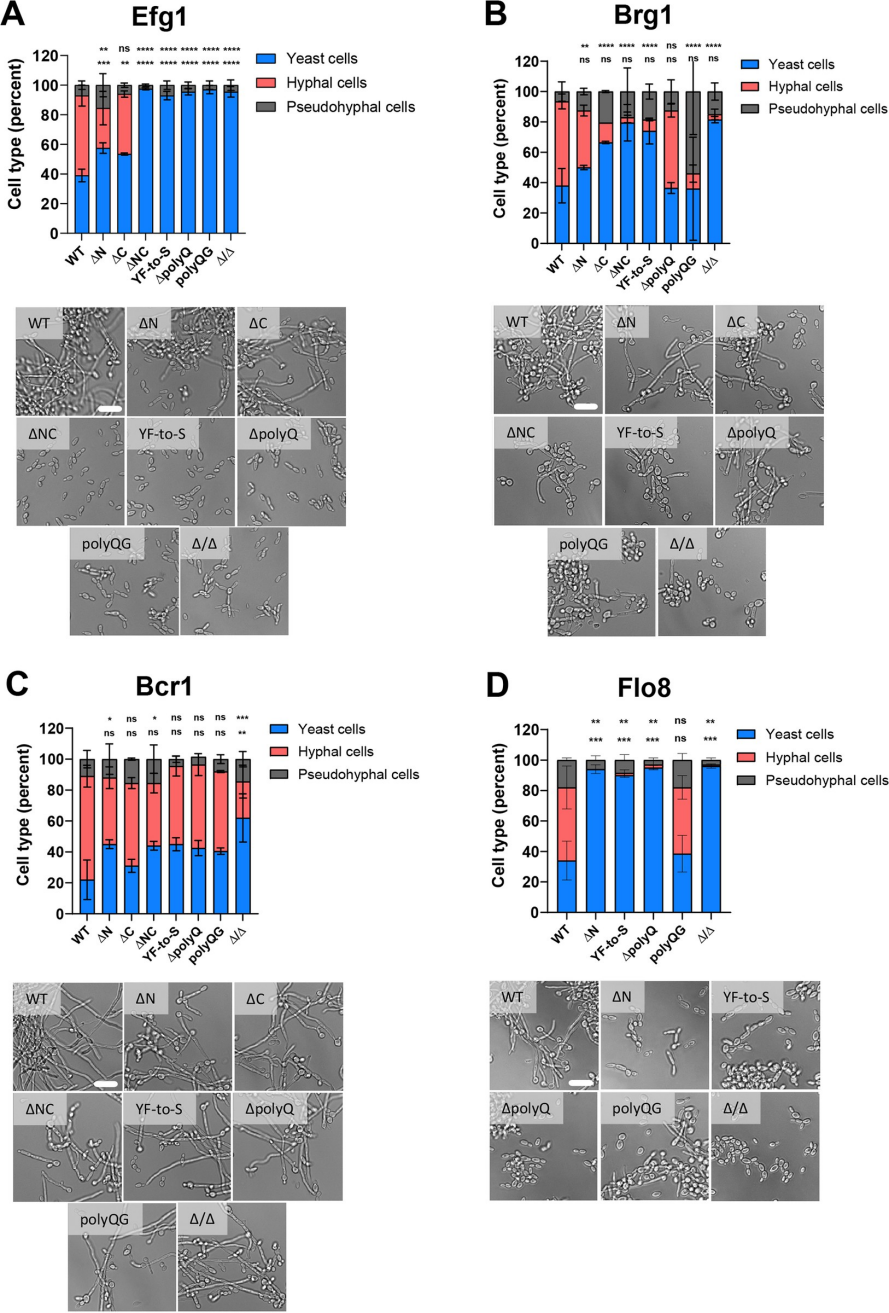
Analysis of the impact of TF PrLD mutations on filamentation. **(A-D)** Strains with Efg1 (**A**), Brg1 (**B**), Bcr1 (**C**), and Flo8 (**D**) mutations were grown under hyphal-inducing conditions for 6 hours and cell morphology determined. Representative microscopy images are included. Mean cell count values are shown and error bars show S.D. Statistical analysis was performed using ordinary one-way ANOVA with Dunnett’s multiple-comparison test, in which the mean cell count values were compared with that for control strains. P values are reported for mean values relative to that for the controls for yeast-form cell counts (bottom row) and hyphal cell counts (top row). Experiments were repeated at least twice, and n = 5 with 20 cells analyzed per image for a total of 200 cells per strain. *P < 0.05; **P < 0.01; ***P < 0.001; ****P < 0.0001; ns = not significant. Scale bars; 10 μm.

In general, TF mutants showed a wide range of filamentation phenotypes. All Efg1 PrLD deletion and amino acid substitution mutants showed a marked filamentation defect. Hyphae formation for single PrLD deletions strains was decreased, while no hyphal cells were detected for the ΔNC mutant or for YF-to-S, ΔpolyQ or polyQG mutants (**Fig 4A**). Despite the loss of hyphal cells, a low percentage of pseudohyphal cells were still formed by these Efg1 mutants.

For Brg1, hyphal cell formation was significantly reduced for all mutants tested except for the ΔpolyQ derivative, while the polyQG mutant showed an increased tendency to form pseudohyphal cells instead of hyphal cells (**Fig 4B**). We note that the Brg1 ΔpolyQ mutant exhibited a higher biofilm mass than the YF-to-S and polyQG mutants (**Fig 2**), and thus the propensity to form hyphal cells correlates with the ability to form biofilms for Brg1 mutants.

Most Bcr1 variants were still able to form hyphal cells similar to the control strain. Only the ΔN and ΔNC deletion strains showed significant decreases in hyphal cell formation (**Fig 4C**). Previous studies have reported that the role of Bcr1 in *C*. *albicans* biofilms relates to its ability to promote adherence [44–46] and our data is consistent with Bcr1 derivatives being defective in biofilm formation due to adherence defects rather than an inability to filament.

Deletion of the single PrLD in Flo8 caused a significant loss in hyphae formation and a corresponding increase in yeast-form cells (**Fig 4D**). The YF-to-S and ΔpolyQ Flo8 variants also essentially abolished hyphal cell formation (**Fig 4D**). Interestingly, Flo8 polyQG cells were still able to filament and form true hyphae and pseudohyphae (**Fig 4D**) yet were defective in biofilm formation (**Fig 2**). This suggests that Flo8 polyQG cells are defective in biofilm formation due to a characteristic distinct from their propensity to filament.

To evaluate the expression and localization of TF variants, ORFs were C-terminally tagged with mNeonGreen and visualized via microscopy (see **Materials and Methods**). These assays demonstrated that Efg1 and Flo8 mutants localized correctly to the nucleus and did not have significantly less expression than WT controls (**S2 Fig**), indicating that phenotypes were not the result of changes in expression. Bcr1 and Brg1 did not express at high enough levels to visualize by microscopy, and western blotting was therefore used to establish that Bcr1 variants were expressed at similar levels to the wildtype protein (**S3 Fig**). Brg1 protein was undetectable even by western blot, although the YF-to-S mutant showed comparable RNA expression to the WT control (see RNA analysis below).

Overall, these results establish that the PrLDs of biofilm TFs have critical roles in regulating *C*. *albicans* filamentation, and that substitution of specific PrLD amino acids can abolish hyphae formation and biofilm formation.

### Biofilm TF YF-to-S mutants exhibit reduced expression of hyphal-specific genes

**Fig 5:**
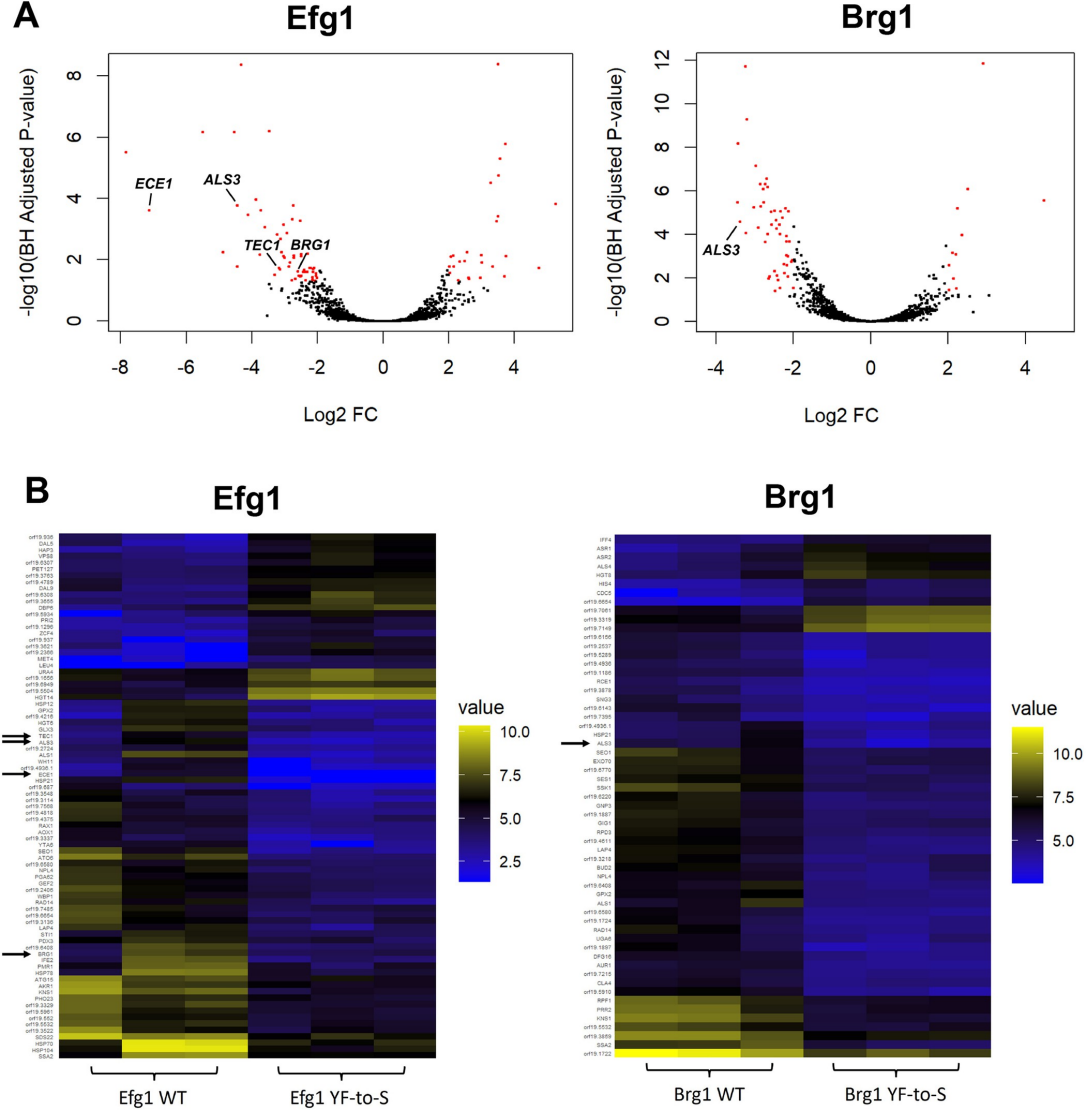
TF YF-to-S mutants decrease hyphal-specific gene expression. **(A)** Volcano plots of differentially expressed genes between WT and YF-to-S mutants of Efg1 and Brg1 (in triplicate) with significantly upregulated or downregulated genes in red (up or down in YF-to-S mutants compared to WT). Several key master biofilm TFs and hyphal-specific genes are highlighted. **(B)** Heat maps of differentially expressed genes (>4 fold) between WT and YF-to-S mutants for Efg1 and Brg1, with arrows highlighting master biofilm TFs or key hyphal-specific genes. Benjamini-Hochberg adjusted *P* value < 0.05.

RNA sequencing on Efg1 and Brg1 WT and YF-to-S mutants was performed to examine the impact of these mutations on gene expression under filament-inducing conditions (Spider medium at 37°C for 6 hours). As noted above, the Efg1 YF-to-S mutant showed a complete inability to form hyphal filaments under these conditions whereas the Brg1 YF-to-S mutant showed a large reduction, but not a complete block, in hyphae formation (**Fig 4A** and **4B**). 82 genes were differentially expressed >4-fold (adjusted *P* value < 0.05) between WT and YF-to-S Efg1 strains (29 genes up and 53 genes down in the mutant relative to the WT). Differentially regulated GO (gene ontology) processes included biofilm formation, proteolysis, and heat acclimation (**Fig 5 and S1 Appendix**). In comparison, 59 genes were differentially expressed between WT and YF-to-S Brg1 strains (11 genes up and 48 genes down in the mutant relative to the WT), with the only differentially regulated GO process being interspecies interaction (**Fig 5 and S1 Appendix**). Interestingly, Efg1 YF-to-S mutants failed to express two master biofilm TFs, *BRG1* and *TEC1*, as well as two key filamentation-specific genes, *ALS3* and *ECE1* [42], to the same degree as the WT strain. Of these four genes, Brg1 YF-to-S mutants only showed an inability to induce *ALS3* relative to the WT control (**Fig 5 and S1 and S2 Appendices**). These data demonstrate that removing aromatic residues from TF PrLDs can inhibit the expression of key filamentation genes and that Efg1 mutations had a larger impact than Brg1 mutations on the expression of other master biofilm TFs, in line with the bigger impact of these mutations on filamentation.

### Efg1, Brg1, and Flo8 PrLDs promote phase separation in live cell nuclei

**Fig 6:**
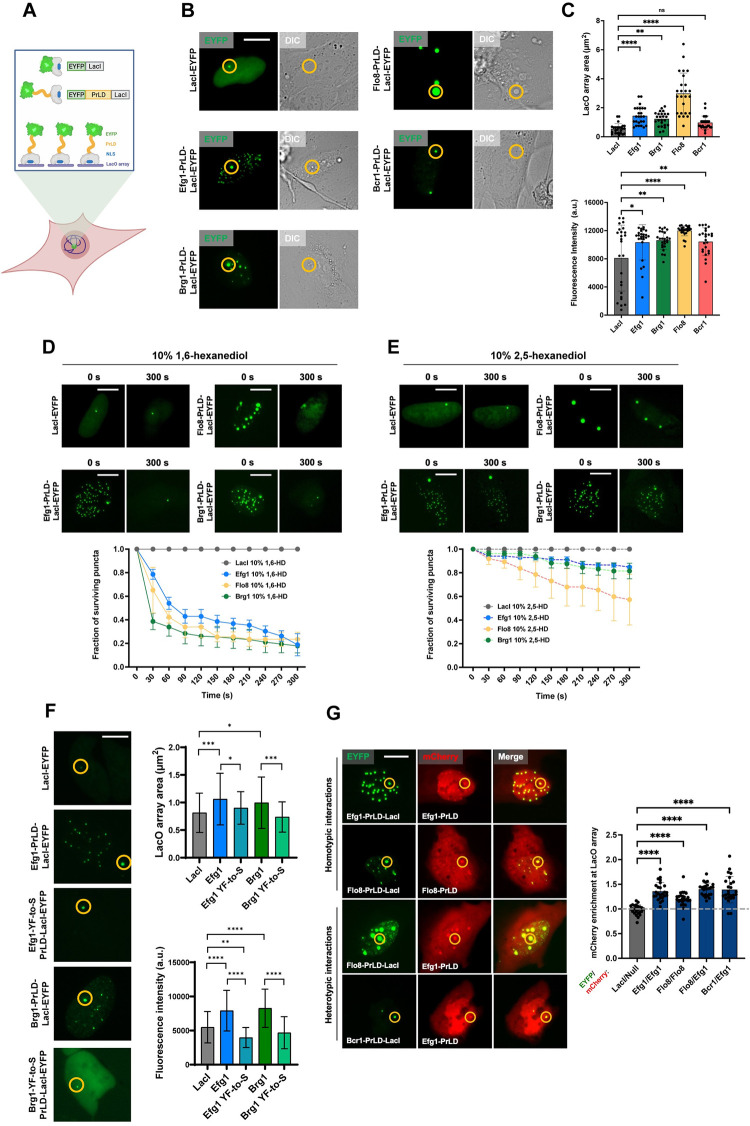
Biofilm TFs form liquid-like, phase-separated condensates in U2OS cells. **(A)** Schematic illustrating the LacO array in U2OS cells used to recruit LacI and LacI-PrLD fusion proteins. Created with BioRender.com. **(B)** Representative images of U2OS cells containing a LacO array (shown in yellow circle) and expressing a LacI-EYFP control, or Efg1, Brg1, Flo8, or Bcr1 PrLDs fused to LacI-EYFP. Scale bar; 10 μm. **(C)** Quantification of average size (top) and average fluorescence intensity (bottom) of the LacO array bound by the LacI-EYFP control or PrLD constructs. Values show the mean area and fluorescence intensity at the LacO array, and error bars show S.D. **(D,E)** Representative images of cells before and after treatment with 10% 1,6-HD (**D**) or 10% 2,5-HD (**E**). Scale bars; 10 μm. Graphs track number of surviving puncta over time. Error bars show S.E.M. **(F)** Left side: representative images of U2OS cells expressing a LacI-EYFP control or Efg1 or Brg1 (WT or YF-to-S mutant) PrLDs fused to LacI-EYFP. Yellow circles indicate LacO array. Scale bar; 10 μm. Right side: quantification of average size (top) and fluorescence intensity (bottom) of the LacO array bound by LacI constructs. Values show the mean and error bars show S.D. **(G)** Representative images of *C*. *albicans* TF PrLD-LacI-EYFP and PrLD-mCherry constructs co-expressed in U2OS LacO cells (left) and quantification of mCherry signal enrichment at the LacO array (right). Enrichment is defined as maximum mCherry intensity at the array divided by average intensity outside of array. Null construct is mCherry alone. Enrichment greater than 1 indicates PrLD-PrLD interactions at the array. Mean enrichment values are shown, and error bars are S.D. All statistical analyses were performed using ordinary one-way ANOVA with Dunnett’s multiple-comparison test, in which the mean enrichment value was compared with that for the control Null/LacI construct. P-values are reported for mean values relative to that for the Null/LacI control. Experiments were repeated at least twice with similar results, and n = 20–25 with images analyzed for 25 individual cells per construct. *P < 0.05; **P < 0.01; ***P < 0.001; ****P < 0.0001. Scale bar; 10 μm.

The potential for *C*. *albicans* TF PrLDs to form biomolecular condensates was evaluated by expression in a mammalian U2OS cell line. This cell line contains ~50,000 copies of the Lac operator (LacO) array integrated into the genome and has been used to demonstrate liquid-liquid phase separation (LLPS) of both human and yeast TFs [14,28,35,47]. In this system, Lac repressor (LacI) is recruited to the LacO array and can be used to visualize the localization of TF PrLDs fused to LacI-EYFP in live cells (**Fig 6A**) [35,47]. A LacI-EYFP control forms a single spot at the array whereas phase separation of TF-LacI-EYFP fusion proteins generates larger foci at the array, often with additional puncta throughout the nucleus [14,28,35].

Efg1 PrLDs were previously shown to form phase-separated condensates in U2OS cells and, consistent with this, a larger, brighter LacO array spot was evident in cells expressing Efg1-LacI-EYFP compared to LacI-EYFP alone (**Fig 6B and 6C**)[28]. Brg1- and Flo8-LacI-EYFP constructs similarly formed significantly larger LacO-associated foci than the LacI-EYFP control (**Fig 6B and 6C**). Foci formed by PrLD-LacI fusions were also visible by differential interference contrast (DIC) microscopy, indicating their refractive index and mass density is significantly different from the surrounding cellular environment (**Fig 6B**) [35]. In addition to array-associated foci, Efg1, Brg1, and Flo8 PrLDs produced additional foci throughout the nuclei (**Fig 6B**). In contrast, Bcr1-LacI-EYFP formed foci only at the LacO array and these foci were not significantly larger than those of the control (**Fig 6B and 6C**), suggesting that Bcr1 PrLDs have a lower ability to promote phase-separation events.

The aliphatic alcohol 1,6-hexanediol (1,6-HD) preferentially disrupts weak hydrophobic interactions in condensates with liquid-like properties, but has little effect on gel-like or solid assemblies [48–50]. To examine the physical properties of TF puncta, U2OS cells were treated with 10% 1,6-HD for 5 minutes which caused Efg1, Brg1, and Flo8 condensates to rapidly dissolve, while the LacI-EYFP control signal was unaffected (**Fig 6D**). The compound 2,5-hexanediol (2,5-HD) is closely related to 1,6-HD but does not dissolve liquid condensates as efficiently [35]. Treatment with 10% 2,5-HD did not impact Efg1, Brg1, or Flo8 condensates to the same extent as 1,6-HD, consistent with these droplets having liquid-like droplet properties (**Fig 6E**).

YF-to-S derivatives of Efg1 and Brg1 PrLDs were also tested in U2OS cells to assess phase separation capabilities. Neither YF-to-S mutant formed enlarged/brighter foci at the LacO array (**Fig 6F**). Furthermore, neither mutant formed additional condensates outside of LacO foci. These results indicate that while PrLDs can increase the proclivity for phase separation, key residues (specifically aromatic residues) within these domains are critical for promoting phase separation events.

### PrLDs enable homotypic and heterotypic interactions between TFs

The ability of PrLDs to form condensates in U2OS cells prompted us to test whether these domains can mediate homotypic or heterotypic interactions. TF PrLDs were fused to LacI-EYFP (as above) or to mCherry and co-expressed in U2OS cells containing the LacO array. Co-localization of EYFP and mCherry signals at the LacO array was then used as a readout of PrLD-dependent interactions.

PrLDs from Efg1 and Flo8 both underwent homotypic (self) interactions as evidenced by the significant overlap of EYFP and mCherry signals when fused to these PrLDs as compared to controls (**Fig 6G**). PrLD-mediated co-localization was seen not only at the LacO array but also at multiple other loci throughout the nuclei (**Fig 6G**). The Efg1-PrLD also underwent heterotypic interactions with other TF PrLDs as the Efg1 mCherry signal was recruited to Flo8- and Bcr1-LacI-EYFP foci (**Fig 6G**).

These results indicate that most, but not all, biofilm TF PrLDs can form phase-separated condensates in the cell. They also show that these PrLDs can mediate interactions between TFs in the biofilm TRN, including those involving Efg1. Interestingly, protein-protein interactions can be mediated by PrLDs even in the absence of enlarged condensate foci, as the small foci formed by Bcr1 at the LacO array were still capable of recruiting Efg1.

### Biofilm TFs undergo phase separation *in vitro* and these condensates can incorporate the C-terminal domain of RNA polymerase II

**Fig 7:**
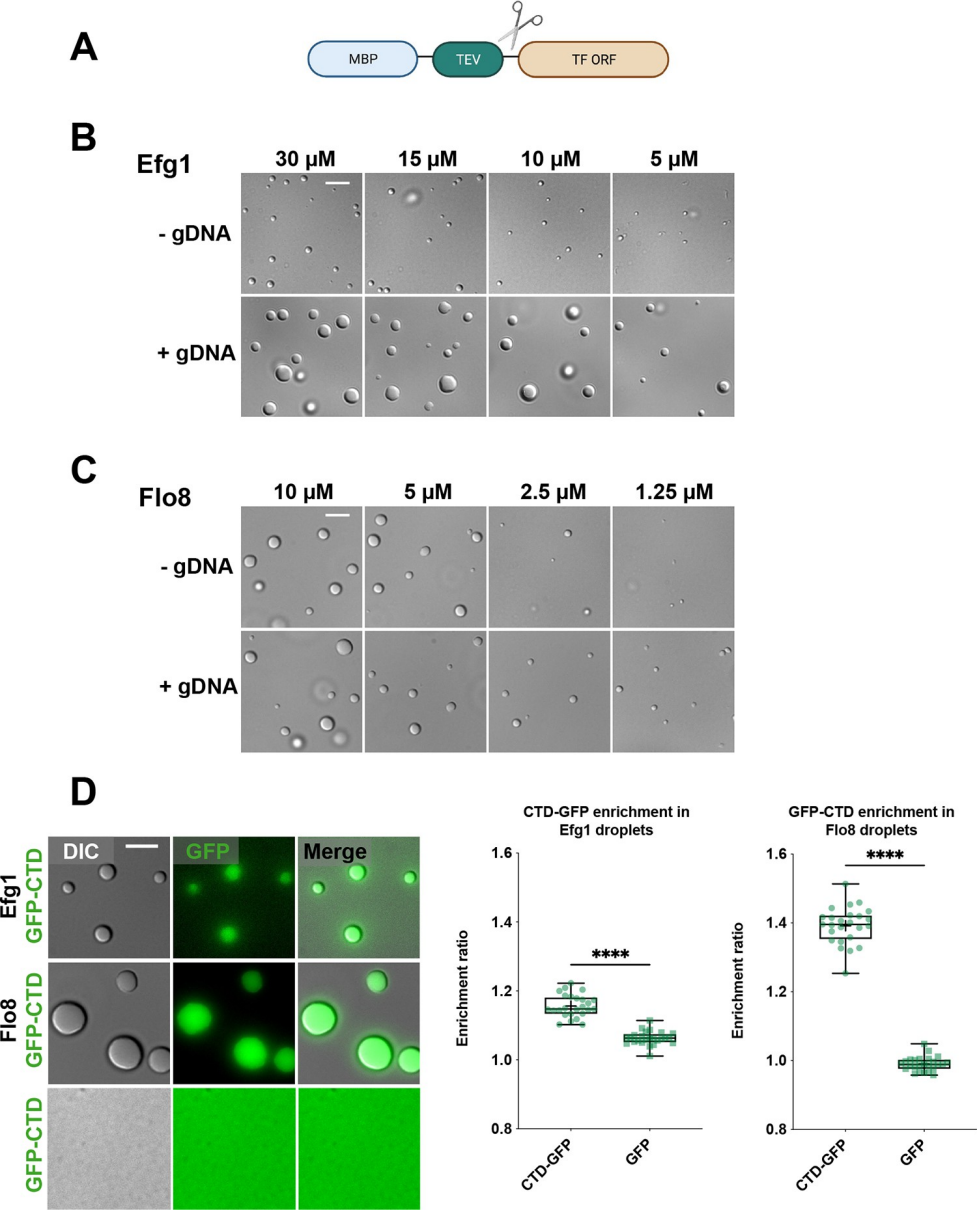
Efg1 and Flo8 form phase-separated condensates *in vitro*. **(A)** Schematic of vector for recombinant protein purification. MBP, maltose binding protein; TEV, tobacco etch virus protease site; TF ORF, transcription factor open reading frame. Created with BioRender.com. **(B,C)** Representative images of protein droplets formed by Efg1 (**B**) and Flo8 (**C**) with and without *C*. *albicans* gDNA. Proteins were treated with TEV protease for 30 min at 22°C in 10 mM Tris-HCl buffer with 150 mM NaCl. Flo8 buffer also included 5% PEG-8000. gDNA was included at a final concentration of 50 nM. Images represent single experimental replicates, with experiments repeated at least twice. Scale bars; 5 μm. **(D)** Representative images of Efg1 and Flo8 protein droplets with addition of RNA Pol II GFP-CTD, or GFP-CTD alone (left) and quantification of GFP-CTD enrichment in TF droplets (right). Efg1 or Flo8 was mixed with GFP-CTD or GFP and the mixture treated with TEV protease for 30 min at 22°C in 10 mM Tris-HCl buffer with 150 mM NaCl and 5% PEG-8000. Efg1 or Flo8 were included at 15 μM final concentration and GFP-CTD at 1.5 μM final concentration. For quantification, droplets were located in the DIC channel, and intensity for the GFP signal inside the droplet compared to the intensity signal outside the droplet, after subtracting fluorescent background. At least 5 images were used for quantification and 25 total droplets measured for each TF. Box and whisker plots show all data points, maximum to minimum, and indicate enrichment ratios for GFP-CTD in Efg1 or Flo8 droplets. For each plot, data are median (line), mean (‘+’), 25–75th percentiles (box), and 5–95th percentiles (whiskers). Statistical significance was performed using a two-tailed Mann-Whitney U-test. ****P < 0.0001. Scale bar; 5 μm.

The biochemical properties of Efg1 and Flo8 were examined by purification of these proteins from *E*. *coli*. TFs were expressed as fusion proteins with maltose binding protein (MBP) to enhance solubility and treated with TEV protease to release the native TFs from MBP (**Fig 7A**). Purified Efg1 and Flo8 readily formed liquid-like condensates (**Fig 7B and 7C**) with Efg1 forming liquid droplets at concentrations as low as 5 μM even without the inclusion of molecular crowding agents (**Fig 7B**), as previously reported [28]. Purified Flo8 protein also formed liquid condensates, although this required the presence of 5% polyethylene glycol (PEG) as a crowding agent (**Fig 7C**). Liquid-like behavior was evident as droplets undergoing successive fusion events when monitored by microscopy. Nucleic acids can promote certain phase separation events [22,51,52] and we therefore tested the impact of *C*. *albicans* genomic DNA (gDNA) on droplet formation. Inclusion of gDNA resulted in larger Efg1 droplets (**Fig 7C**) in agreement with previous observations [28]. Flo8 droplets were also larger in the presence of gDNA and formed at concentrations as low as 1.25 μM (**Fig 7C**). These results indicate that both Efg1 and Flo8 readily undergo liquid phase separation *in vitro* and that condensation events are promoted by DNA.

RNA polymerase II (RNA pol II) is required for transcription of all protein-encoding genes and most non-coding RNAs [53,54]. RNA pol II contains an intrinsically disordered C-terminal domain (CTD) composed of heptad repeats that are conserved from yeast to humans [55,56]. The CTD has been shown to undergo phase separation and RNA Pol II can form assemblies with other IDR-containing transcriptional regulators [35,55–58]. To test whether the CTD of *C*. *albicans* RNA Pol II could be incorporated into Efg1 and Flo8 droplets, *C*. *albicans* CTD was purified from *E*. *coli* as a fusion protein with MBP-TEV and GFP, and mixed with purified TFs prior to TEV treatment. Upon proteolytic treatment, GFP-CTD was readily recruited into Efg1 and Flo8 droplets whereas GFP alone was not (**Fig 7D**). GFP-CTD itself did not form condensates under our assay conditions (**Fig 7D**). These results indicate that biofilm TFs form phase-separated droplets that can incorporate the intrinsically disordered CTD of *C*. *albicans* RNA pol II.

### PrLD mutations attenuate filamentation during mammalian host colonization

**Fig 8:**
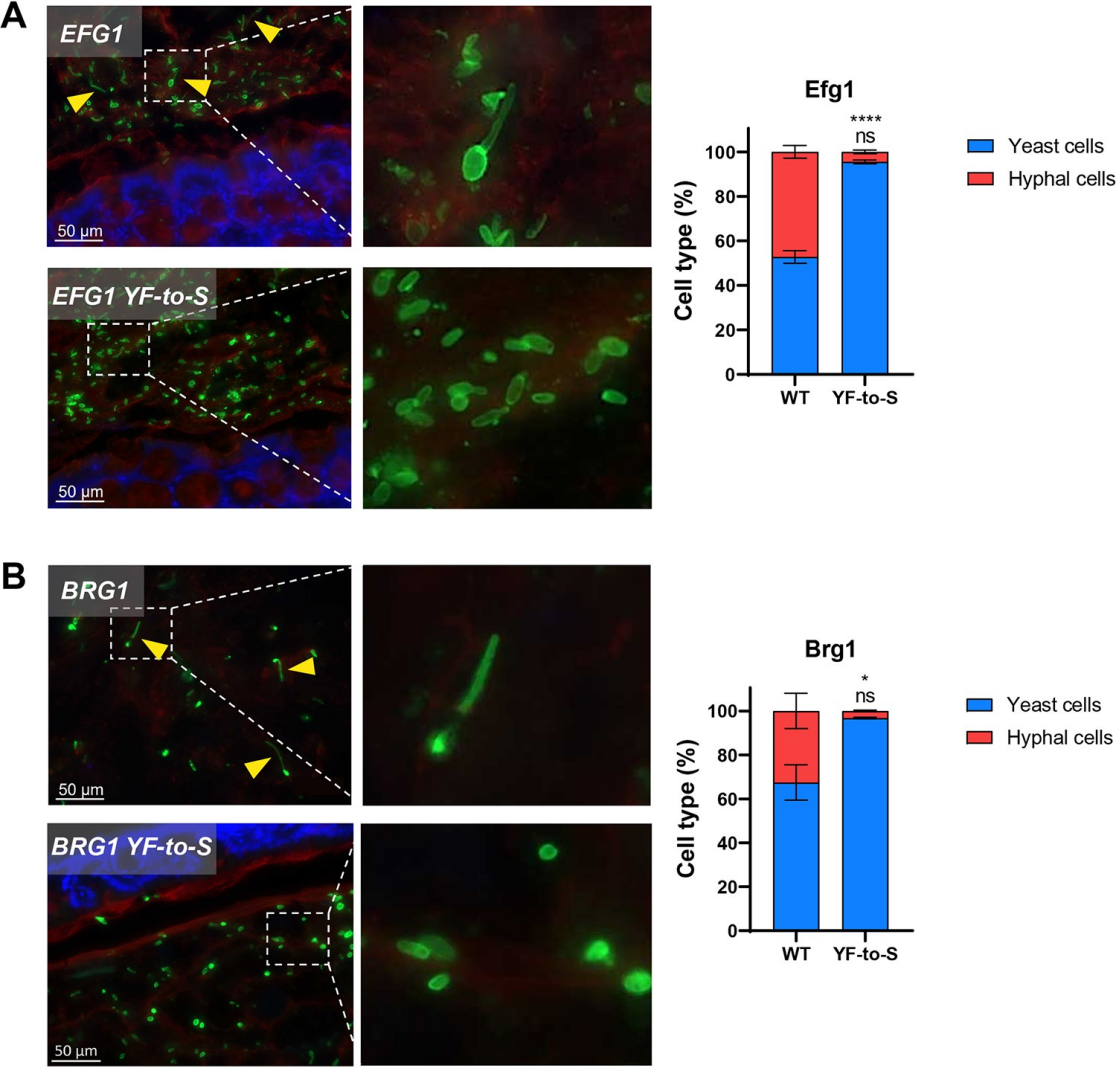
Removal of aromatic amino acids from Efg1 or Brg1 PrLDs blocks *C*. *albicans* filamentation in the mouse gut. **(A,B)** Representative images (left) and cell type counts (right) for mice colonized with the indicated *C*. *albicans* strains, Efg1 (**A**, top) or Brg1 (**B**, bottom). Mice were colonized for 7 days, then colon sections prepared for imaging. In images, *C*. *albicans* cells are green, the intestinal mucus is red, and epithelial cells are blue. Yellow arrows indicate filamentous cells. 2–4 mice were used for each strain, and cell counts combined from pooled colon sections. At least 300 cells were counted per yeast strain. Graphs show cell counts, with error bars indicating S.D. Statistical significance was calculated using a one sample T-test. P values are reported for mean values relative to that for the controls for yeast-form cell counts (bottom row) and hyphal cell counts (top row). *P < 0.05; ****P < 0.0001; ns = not significant.

The data provided above establishes that PrLDs are critical to the function of biofilm TFs for filamentation and biofilm formation under laboratory conditions. However, filamentation of strains in the laboratory does not always reflect their ability to filament in the mammalian host [59,60]. To define the contribution of TF PrLDs to *C*. *albicans* behavior *in vivo*, we evaluated mutant PrLD-expressing strains for their ability to filament during murine GI tract colonization. Mice were pre-treated with antibiotics (to promote fungal colonization) and then gavaged with *C*. *albicans* strains expressing WT or YF-to-S variants of Efg1 or Brg1. Experiments were performed for 7 days and the morphology of fungal cells in the colon examined by staining with an anti-*Candida* antibody (see **Materials and Methods**). Mice colonized with control strains showed a mixture of yeast and hyphal cells consistent with previous studies [59,61–63]. In contrast, YF-to-S mutants of Efg1 and Brg1 formed yeast cells almost exclusively in this niche (**Fig 8A and 8B**). These results establish that PrLD mutations that prevent *C*. *albicans* TFs from forming hyphal cells under laboratory conditions also block the function of these TFs in mammalian gut colonization.

## Discussion

*C*. *albicans* filamentation and biofilm formation are critical for host invasion and device-associated infections. Biofilm development is regulated by a TRN of nine master TFs that bind to key regulatory regions throughout the genome, including positions that often lack consensus binding motifs for one or more of these TFs [11,12]. It is therefore envisaged that physical interactions between biofilm TFs are integral to their function in regulating target gene expression.

In this study, we show that seven of the master biofilm TFs contain intrinsically disordered PrLDs that promote intermolecular interactions and the formation of phase-separated condensates [24,25]. Four biofilm TFs, Bcr1, Brg1, Efg1 and Flo8, were examined in detail and removal of their respective PrLDs completely blocked their ability to support biofilm formation. PrLD removal also inhibited filamentation, with the exception of Bcr1, consistent with previous reports that Bcr1 impacts cell adherence rather than filamentation [11,46]. For Efg1, deletion of subdomains has previously been reported to reduce or abrogate hyphal cell formation [64], in agreement with the data presented here.

Mutational studies of PrLDs have shown that aromatic residues can promote intermolecular interactions and phase separation of proteins via pi-pi or cation-pi interactions [36,37]. We show that YF-to-S substitutions in the PrLDs of Efg1, Bcr1, Brg1 and Flo8 all result in defective TFs, establishing the importance of aromatic residues to their cellular function. Polyglutamine sequences can also influence LLPS, as exemplified in Huntingtin exon 1 protein where longer polyQ tracts are linked to increased phase separation, aggregation and interactions with other LCDs [40,49,65]. PolyQ-ataxin-1, a protein associated with the neurodegenerative disease spinocerebellar ataxia 1, similarly contains an expanded polyQ region that mediates condensate formation [41,66]. In line with these examples, disruption of polyglutamine stretches (ΔpolyQ or polyQG mutants) restricted the function of Efg1, Brg1 and Flo8, but not Bcr1, in biofilm formation.

There was also a clear connection between biofilm and filamentation defects for Efg1 mutants. Efg1 YF-to-S, ΔpolyQ, and polyQG variants were completely defective both for biofilm formation and for filamentation. In contrast, Brg1 ΔpolyQ and Flo8 polyQG mutants were defective in biofilm formation but still formed hyphal filaments in planktonic cultures at frequencies close to those of control strains. These results suggest that Brg1 and Flo8 may impact biofilm development by mechanisms other than filamentation, or that filamentation within biofilms differs from that under planktonic conditions. RNA sequencing of WT and mutant TF strains corroborated the phenotypic results. Thus, Efg1 and Brg1 YF-to-S mutants that had substantial defects in filamentation also displayed defects in hyphal-specific gene expression. Moreover, the Efg 1 mutant showed a more complete loss of hyphae formation than the equivalent Brg1 mutant and had a greater impact on gene expression, especially among hyphal-associated genes.

Two chimeric TFs, Efg1-Taf15 and Flo8-Taf15, were examined for their functionality in which the PrLDs of *C*. *albicans* TFs were replaced with that from the human protein TAF15. Interestingly, the Efg1-Taf15 chimera showed almost wildtype activity in biofilm assays whereas the Flo8-Taf15 protein was essentially non-functional. These results indicate that a certain plasticity exists between PrLDs but also that not all PrLDs are functionally interchangeable. This is clearly an important area for additional investigation to understand the properties of PrLDs that enable them to retain functionality when exchanged between TFs.

The potential for biofilm PrLDs to form phase-separated condensates was examined by expression in mammalian U2OS cells. PrLDs of Efg1, Brg1, and Flo8, but not Bcr1, supported the formation of liquid condensates both at a LacO array and at additional positions throughout the nucleus. These condensates dissolved faster when treated with 1,6-HD than with 2,5-HD indicating that weak hydrophobic interactions contributed to their phase separation [48,50]. Critically, YF-to-S substitutions in Efg1 and Brg1 prevented the formation of condensates (both at the LacO array and elsewhere in the nucleus), indicating that these mutations block both TF function and phase separation. We note that PrLDs could promote homotypic interactions (e.g., Efg1-Efg1 or Flo8-Flo8 interactions) while certain PrLDs were recruited into foci formed by other PrLDs (e.g., Efg1 was recruited to Flo8 or Bcr1 foci) via heterotypic interactions. While we focused on the role of TF PrLDs in phase separation, DNA binding domains may also influence these events given that structured domains/motifs can potentiate protein condensation [22,67], and thus the analysis of full-length TFs in this model could be of interest in the future.

Biochemical assays showed that purified Flo8, like Efg1 [28], readily forms liquid droplets *in vitro*, and that droplet formation is stimulated by DNA. This is in line with other reports of nucleic acids promoting protein condensation events [15, 16, 68]. We also showed that the CTD of *C*. *albicans* RNA Pol II was incorporated into droplets formed by Efg1 or Flo8 but did not readily form droplets by itself. Recent studies have shown that the CTDs of *S*. *cerevisiae* and human RNA Pol II both form liquid condensates (in the presence of molecular crowding agents) which may help initiate gene expression [55, 69]. Our data similarly suggests that PrLD-CTD interactions may be important for recruitment of RNA Pol II to *C*. *albicans* promoters for induction of gene expression. Given that phosphorylation of RNA Pol II can alter its propensity to phase separate [55, 69], it will now be important to also examine the role of post-translational modifications on *C*. *albicans* RNA Pol II and transcription.

Currently, two competing models of transcriptional activation have been proposed in which the transcriptional machinery assembles via the formation of phase-separated condensates or by forming transient “hubs” that do not involve phase-separation events [70,71]. Both models invoke “fuzzy” interactions between TFs, Mediator complex, and RNA pol II, although they differ in whether the components reach the concentration thresholds necessary for phase separation [56,72–74]. Our experiments establish that the forces that promote PrLD-PrLD interactions are critical for TF function in *C*. *albicans*. However, it remains possible that PrLDs are supporting the formation of transient TF hubs rather than more stable phase-separated condensates to regulate *C*. *albicans* gene expression. In this regard, it is notable that Bcr1 PrLDs, unlike those of Efg1, Flo8 or Brg1, do not form condensates in U2OS cells but can still interact with Efg1 PrLDs. We therefore suggest that *C*. *albicans* biofilm TFs may interact via PrLDs both via forming transient hubs and by forming phase-separated condensates depending on the combinations of TFs involved and their concentration levels at different sites in the genome. More experiments will be needed to address these possibilities, something that is hindered by the relatively small size of fungal nuclei compared to those in higher eukaryotes.

Finally, we addressed whether PrLD mutations that abolish TF function under *in vitro* culture conditions similarly impact their function during host infection. This is important as the properties of *C*. *albicans* cells in the host do not always reflect those in culture, emphasizing the necessity to directly evaluate morphology *in vivo* [59,60]. *C*. *albicans* colonizes diverse mammalian niches including the GI tract where it exists as both yeast- and hyphal-form cells [59,75]. We evaluated YF-to-S mutants of Efg1 and Brg1 that are both highly defective for filamentation *in vitro* and established that they are also defective for filamentation during GI colonization. These experiments establish that removal of aromatic residues from TF PrLDs prevents *C*. *albicans* filamentation in the host, a trait which is integral to biofilm formation and pathogenesis [1,59,76].

## Conclusions

A highly interconnected network of TFs regulates filamentation and biofilm formation in *C*. *albicans*. We demonstrate that the PrLDs present in these master TFs are integral to their function as they enable protein-protein interactions including homotypic interactions, heterotypic TF-TF interactions and those with the CTD of RNA Pol II. Disruption of PrLD-PrLD contacts can therefore abolish key traits that are critical to fungal behavior and pathogenesis. A full understanding of PrLD contributions to transcription and phase separation behavior could pave the way for new therapeutic approaches for disabling TRNs, with far reaching consequences not only for the treatment of fungal diseases but also for altering aberrant TRNs such as those associated with human disorders including cancer [77–79].

## Materials and methods

### Plasmid construction

*C*. *albicans* TF constructs were created in pSFS2A [80] or derivatives of this plasmid as indicated below. A plasmid containing full length Efg1, pRB360, was constructed as described [81]. The Efg1 ΔN PrLD plasmid (pRB630) was created via fusion PCR of two fragments amplified from pRB360: (1) Efg1 5’ flank with oligos 1838/3916; and (2) Efg1 ΔN PrLD ORF with oligos 3915/1839. Splicing of the two fragments with overlap extension (SOE)-PCR was conducted with oligos 1838/1839, and the resulting fragment cloned into pSFS2A with ApaI/KpnI [82]. The Efg1 ΔC PrLD plasmid (pRB632) was created with fusion PCR of two fragments amplified from pRB360: (1) Efg1 5’ flank and ΔC ORF with oligos 1838/3918; and (2) remaining Efg1 ΔC ORF and 3’ flank with oligos 3917/1839. Fragments were fused with SOE-PCR and the product cloned into pSFS2A with ApaI/KpnI. The Efg1 ΔNC PrLD plasmid (pRB634) was created with fusion PCR of three fragments amplified from pRB360: (1) Efg1 5’ flank with oligos 1838/3916; (2) Efg1 DNA binding domain with oligos 3915/3918; and (3) Efg1 3’ flank with oligos 3917/1839. Fragments were fused via SOE-PCR and cloned into pSFS2A using ApaI/KpnI.

For Efg1 PrLD amino acid mutants, PrLD sequences were synthesized by BioBasic, and plasmids assembled via Golden Gate Assembly (GGA). A Golden Gate Assembly (GGA)-adapted version of pSFS2A (pRB1397) was generated by annealing oligos 6048/6049 to create a short double-stranded DNA fragment containing two outward facing BsaI sites, and sticky ends allowing ligation into pSFS2A digested with ApaI/XhoI. The Efg1 3’ flank sequence was amplified from *C*. *albicans* SC5314 gDNA with oligos 6422/6423 and cloned into pRB1397 with SacII/SacI to generate pRB1763. The resulting plasmid was then used as the destination vector for all Efg1 PrLD amino acid mutant GGA reactions. The Efg1 YF-to-S PrLD construct (pRB1610) was created via GGA of four PCR fragments: (1) Efg1 5’ flank from gDNA with oligos 6376/6377; (2) Efg1 N-terminal YF-to-S PrLD from pRB1858 with oligos 6378/6379; (3) Efg1 DNA binding domain from gDNA with oligos 6380/6381; and (4) Efg1 C-terminal YF-to-S PrLD from pRB1858 with oligos 6382/6383. Fragments were assembled via GGA with BsaI-HFv2 (NEB). The Efg1 ΔpolyQ PrLD construct (pRB1612) was created with GGA of four PCR fragments: (1) Efg1 5’ flank from gDNA with oligos 6376/6391; (2) Efg1 N-terminal ΔpolyQ PrLD from pRB1860 with oligos 6392/6393; (3) Efg1 DNA binding domain from gDNA with oligos 6394/6395; and (4) Efg1 C-terminal ΔpolyQ PrLD from pRB1860 with oligos 6396/6397. Fragments were assembled by GGA reaction with BsaI-HFv2. The Efg1 polyQG PrLD construct (pRB1611) was created via GGA of four PCR fragments: (1) Efg1 5’ flank from gDNA with oligos 6376/6384; (2) Efg1 N-terminal polyQG PrLD from pRB1859 with oligos 6385/6386; (3) Efg1 DNA binding domain from gDNA with oligos 6387/6388; and (4) Efg1 C-terminal polyQG PrLD from pRB1859 with oligos 6389/6390. Fragments were assembled by GGA with BsaI-HFv2. The Efg1-Taf15 plasmid (pRB1946) was created via GGA with 4 pieces: (1) Efg1 5’ flank was amplified from gDNA with oligos 6376/6377; (2) Taf15 PrLD was amplified from pRB1210 with oligos 7771/7772; (3) The Efg1 DBD was amplified from gDNA with oligos 7775/7776; (4) Taf15 IDR was amplified from pRB1210 with oligos 7773/7774. Fragments were assembled into the vector by GGA with BsaI-HFv2.

Brg1 and PrLD mutant derivatives were made via PCR and cloned into pSFS2A. The backbone for all of these constructs included the Brg1 3’ flank, amplified from gDNA with oligos 6103/6104, then cloned into pSFS2A with SacII/SacI. For the full-length construct (pRB1601), the *BRG1* ORF (with 5’ flank) was amplified from gDNA with oligos 6099/6102, then cloned into the pSFS2A vector with KpnI/ApaI. For the Brg1 ΔN PrLD plasmid (pRB1602), two fragments were amplified from gDNA: (1) 5’ flank with ORF (no N-terminal PrLD) with oligos 6099/6113; and (2) remainder of the ORF with oligos 6112/6102. SOE-PCR was used to fuse fragments together, and the resulting product was digested with KpnI/ApaI and cloned into pSFS2A. For the Brg1 ΔC PrLD plasmid (pRB1603), the ORF without the C-terminal PrLD was amplified from gDNA with oligos 6099/6105, and the fragment cloned into the pSFS2A vector with KpnI/ApaI. The Brg1 ΔNC PrLD plasmid (pRB1604) was created via fusion PCR of two fragments: (1) 5’ flank with ORF (no N-terminal PrLD) using oligos 6099/6113; and (2) remainder of ORF (no C-terminal PrLD) with oligos 6112/6105. Fragments were stitched together via SOE-PCR, and the product cloned into pSFS2A with ApaI/KpnI.

For the Brg1 PrLD mutants, PrLD sequences were synthesized by BioBasic and plasmids assembled via GGA using the modified pSFS2A plasmid with the Brg1 3’ flank, which was amplified from gDNA with oligos 6103/6104 and cloned into pRB1397 with SacII/SacI. The Brg1 YF-to-S PrLD plasmid (pRB1739) was made via GGA of four fragments: (1) Brg1 5’ flank amplified from gDNA with oligos 6569/6570; (2) YF-to-S N-terminal PrLD amplified from pRB1862 with oligos 6571/6572; (3) Brg1 DNA binding domain amplified from gDNA with oligos 6573/6574; and (4) YF-to-S C-terminal PrLD amplified from pRB1862 with oligos 6575/6576. Fragments were assembled via GGA with BsaI-HFv2. The Brg1 ΔpolyQ PrLD plasmid (pRB1740) was made via GGA of four fragments: (1) Brg1 5’ flank amplified from gDNA with oligos 6569/6570; (2) ΔpolyQ N-terminal PrLD amplified from pRB1864 with oligos 6571/6572; (3) Brg1 DNA binding domain amplified from gDNA with oligos 6573/6614; and (4) ΔpolyQ C-terminal PrLD amplified from pRB1864 with oligos 6615/6583. Fragments were assembled by GGA with BsaI-HFv2. The Brg1 polyQG PrLD plasmid (pRB1741) was made via GGA of four fragments: (1) Brg1 5’ flank amplified from gDNA with oligos 6569/6570; (2) polyQG N-terminal PrLD amplified from pRB1863 with oligos 6571/6577; (3) Brg1 DNA binding domain amplified from gDNA with oligos 6573/6578; and (4) polyQG C-terminal PrLD amplified from pRB1863 with oligos 6579/6580. Fragments were assembled by GGA with BsaI-HFv2.

All Bcr1 plasmids were constructed by GGA. The backbone plasmid for the wildtype gene and PrLD mutants was the modified pSFS2A GGA plasmid with the Bcr1 3’ flank sequence, which was amplified from gDNA with oligos 6095/6096 and cloned into pRB1397 with SacII/SacI. For the full length Bcr1 plasmid (pRB1742), the *BCR1* ORF (with 5’ flank) was amplified from gDNA with oligos 6622/6625 and the resulting product assembled by GGA with BsaI-HFv2. The Bcr1 ΔN PrLD plasmid (pRB1743) was assembled from two PCR fragments: (1) Bcr1 5’ flank amplified from gDNA with oligos 6622/6623; and (2) the *BCR1* ORF (no N-terminal PrLD) amplified from gDNA with oligos 6624/6625. Fragments were assembled by GGA reaction with BsaI-HFv2. The Bcr1 ΔC PrLD plasmid (pRB1744) was assembled from two PCR fragments: (1) Bcr1 5’ flank amplified from gDNA with oligos 6626/6627; and (2) the *BCR1* ORF (no C-terminal PrLD) was amplified from gDNA with oligos 6628/6629. Fragments were assembled by GGA with BsaI-HFv2. The Bcr1 ΔNC PrLD plasmid (pRB1745) was assembled from three PCR fragments: (1) Bcr1 5’ flank amplified from gDNA with oligos 6630/6631; (2) the *BCR1* ORF (no N-terminal PrLD) amplified from gDNA with oligos 6632/6633; and (3) the *BCR1* ORF (no C-terminal PrLD) amplified from gDNA with oligos 6634/6635. Fragments were assembled by GGA with BsaI-HFv2.

For the Bcr1 PrLD amino acid mutant plasmids, PrLD sequences were synthesized by BioBasic. The Bcr1 YF-to-S PrLD plasmid (pRB1746) was made via GGA of five fragments: (1) Bcr1 5’ flank amplified from gDNA with oligos 6863/6864; (2) YF-to-S N-terminal PrLD amplified from pRB1865 with oligos 6865/6866; (3) Bcr1 DNA binding domain amplified from gDNA with oligos 6867/6868; (4) YF-to-S C-terminal PrLD amplified from pRB1865 with oligos 6869/6870; and (5) the remaining *BCR1* ORF amplified from gDNA with oligos 6871/6872. Fragments were assembled by GGA with BsaI-HFv2. The Bcr1 ΔpolyQ PrLD plasmid (pRB1747) was made via GGA of five fragments: (1) Bcr1 5’ flank amplified from gDNA with oligos 6873/6874; (2) ΔpolyQ N-terminal PrLD amplified from pRB1864 with oligos 6875/6876; (3) Bcr1 DNA binding domain amplified from gDNA with oligos 6877/6878; (4) ΔpolyQ C-terminal PrLD amplified from pRB1864 with oligos 6879/6880; and (5) the remaining *BCR1* ORF amplified from gDNA with oligos 6881/6882. Fragments were assembled by GGA with BsaI-HFv2. The Bcr1 polyQG PrLD plasmid (pRB1748) was made via GGA of five fragments: (1) Bcr1 5’ flank amplified from gDNA with oligos 6883/6884; (2) polyQG N-terminal PrLD amplified from pRB1866 with oligos 6885/6886; (3) Bcr1 DNA binding domain amplified from gDNA with oligos 6887/6888; (4) ΔpolyQ C-terminal PrLD amplified from pRB1866 with oligos 6889/6890; and (5) the remaining *BCR1* ORF amplified from gDNA with oligos 6891/6892. Fragments were assembled by GGA with BsaI-HFv2.

All Flo8 constructs were created via PCR and cloned into the pSFS2A backbone. The backbone included the Flo8 3’ flank, amplified from gDNA with oligos 6089/6090, then cloned into pSFS2A with SacII/SacI. For the full length Flo8 plasmid (pRB1790), the *FLO8* ORF (with 5’ flank) was amplified from gDNA with oligos 6085/6088, and the product cloned into pSFS2A with ApaI/XhoI. The Flo8 ΔN PrLD DNA sequence was synthesized by Twist BioScience. The Flo8 ΔN PrLD plasmid (pRB1791) was made via fusion PCR of two fragments: (1) Flo8 5’ flank amplified from gDNA with oligos 6085/6086; and (2) Flo8 ΔN PrLD amplified from pRB1871 with oligos 6108/6088. The products were fused together with SOE-PCR and the fragment cloned into the pSFS2A vector with ApaI/XhoI. The Flo8 PrLD amino acid mutants were synthesized by BioMatik. The Flo8 YF-to-S PrLD plasmid (pRB1793) was made via PCR, with the YF-to-S PrLD (including 5’ flank) amplified from pRB1867 with oligos 6085/6088, then cloned into the pSFS2A vector with ApaI/XhoI. The Flo8 ΔpolyQ PrLD plasmid (pRB1794) was made via PCR, with the ΔpolyQ PrLD (including 5’ flank) amplified from pRB1868 with oligos 6085/6088, then cloned into the pSFS2A vector with ApaI/XhoI. The Flo8 polyQG PrLD plasmid (pRB1795) was made via PCR, with the polyQG PrLD (including 5’ flank) amplified from pRB1869 with oligos 6085/6088, then cloned into the pSFS2A vector with ApaI/XhoI. The Flo8-Taf15 plasmid (pRB2042) was assembled with GGA with 3 pieces using the pSFS2A vector with Flo8 3’ flank as the backbone. (1) Flo8 5’ flank and N terminal region were amplified from gDNA with oligos 7777/7780; (2) Taf15 IDR was amplified from pRB1210 with oligos 7783/7772; (3) Flo8 C terminal region was amplified from gDNA with oligos 7781/7782. Fragments were assembled by GGA with BsaI-HFv2.

For protein assays, *EFG1* and *FLO8* ORFs were codon-optimized for expression in *E*. *coli*. The synthetic ORFs were then cloned into plasmid pRP1B–MBP/THMT (pRB523) with restriction enzymes NdeI/XhoI to create plasmids pRB514 and pRB971, respectively [83, 84]. The GFP-CTD of RNA Pol II was created via fusion PCR of 2 fragments: (1) GFP was amplified from pRB690 with oligos 4877/4878; and (2) the C-terminal domain of RNA Pol II was amplified from pRB984 (codon-optimized for *E*. *coli* expression, synthesized by Biomatik) with oligos 5084/5085. SOE PCR was carried out to fuse the two fragments with oligos 4877/5085. The resulting product was cloned into pRB523 with restriction enzymes NheI/XhoI to generate pRB1034. The pMBP–GFP plasmid (pRB723) was created by PCR amplifying GFP from pRB690 (oligos 4122/4123), which was cloned into pRB523 with NheI/XhoI.

For expression of *C*. *albicans* TF PrLDs in U2OS LacO cells, as either LacI-EYFP or mCherry fusions, plasmids were constructed with codon-optimized sequences for expression in *E*. *coli*. The Efg1-PrLD-LacI-EYFP plasmid (pRB1222) was constructed previously [28]. The Flo8-PrLD-LacI-EYFP plasmid (pRB1262) was constructed by amplifying the *FLO8* PrLD from pRB960 using oligos 5680/5681. The resulting insert was digested and cloned into pRB1208 with BsrGI/BspEI. The Brg1-PrLD-LacI-EYFP (pRB1595) construct was created via a fusion PCR of three fragments: (1) the N-terminal PrLD of Brg1 was amplified from pRB832 with oligos 6502/6503; (2) EYFP was amplified from pRB1208 with oligos 6518/6519; (3) the C-terminal PrLD of Brg1 was amplified from pRB832 with oligos 6504/6505. SOE PCR was carried out with oligos 6502/6505. The fragment was digested with SpeI/BspEI and cloned into pRB1208 digested with NheI/BspEI (SpeI and NheI yield compatible sticky ends for ligation reaction). The Bcr1-PrLD-LacI-EYFP plasmid (pRB1597) was created via a three-way fusion PCR: (1) the Bcr1 N-terminal PrLD was amplified from pRB841 with oligos 6510/6511; (2) EYFP was amplified from pRB1208 with oligos 6518/6519; (3) the Bcr1 C-terminal PrLD was amplified from pRB841 with oligos 6512/6513. SOE PCR with oligos 6510/6513 yielded a fusion product that was digested with NheI/BspEI and cloned into pRB1208.

The Efg1 YF-to-S PrLD-LacI-EYFP plasmid (pRB2046) was created by GGA with pSFS2a using: (1) Efg1 YF-to-S N PrLD amplified from pRB1789 with oligos 7897/7898; (2) EYFP amplified from pRB1222 with oligos 7899/7825; (3) Efg1 YF-to-S C PrLD amplified from pRB1789 with oligos 7826/7900. Fragments were assembled by GGA with BsaI-HFv2. The cassette was amplified by PCR with oligos 8030/8031, digested with BspEI and NheI-HF and cloned into pRB1208. The Brg1 YF-to-S PrLD-LacI-EYFP plasmid (pRB2045) was created by GGA with pSFS2a using: (1) Brg1 YF-to-S N PrLD amplified from pRB1785 with oligos 7746/7747; (2) EYFP amplified from pRB1222 with oligos 7744/7745; (3) Brg1 YF-to-S C PrLD amplified from pRB1785 with oligos 7806/7807. Fragments were assembled by GGA with BsaI-HFv2. The cassette was amplified by PCR with oligos 8028/8029, digested with AgeI and BspEI and cloned into pRB1208.

The Efg1-PrLD-mCherry plasmid (pRB1224) was constructed as described [28]. The Flo8-PrLD-mCherry plasmid (pRB1264) was created via PCR amplification of the Flo8 PrLD from pRB960 using oligos 5680/5682. The resulting product was cloned into pRB1207 with BsrGI/BspEI.

### *C*. *albicans* strain construction

Plasmids containing WT *EFG1* or those with PrLD deletions were digested with a unique HpaI site for targeting to the endogenous *EFG1* locus and transformed using the lithium acetate/PEG/heat shock method. Plasmids were integrated into an *efg1Δ/Δ* strain (CAY3009) to yield strains: Efg1 WT (CAY9725); Efg1 ΔN PrLD (CAY9728); Efg1 ΔN PrLD (CAY9730); Efg1 ΔNC PrLD (CAY9732); and Efg1-Taf15 (CAY13647). Integration of constructs was confirmed by PCR with oligos 1840/2933. Efg1 PrLD amino acid mutant plasmids were digested with ApaI/SacI and integrated into the endogenous *EFG1* locus. Plasmids were integrated into an *efg1Δ/Δ* strain (CAY3009) to yield strains: Efg1 YF-to-S PrLD (CAY11947); Efg1 ΔpolyQ PrLD (CAY11949); and Efg1 polyQG (CAY11948). Strains carrying a second allele of WT *EFG1* (CAY12633) or the YF-to-S mutant (CAY14163) were created by recycling the *SAT1* cassette from their respective single addback strains (CAY9725 and CAY11947) and integrating the plasmid at the second allele. Strains were checked by PCR with oligos 1838/4438 (5’ junction) and 4439/6458 (3’ junction).

The WT Brg1 plasmid, all Brg1 PrLD deletion plasmids, and all Brg1 PrLD amino acid mutant plasmids were digested with KpnI/SacI and integrated into the endogenous *BRG1* locus. Plasmids were integrated into a *brg1Δ/Δ* strain (CAY3004) to yield strains: Brg1 WT (CAY11942); Brg1 ΔN PrLD (CAY11943); Brg1 ΔC PrLD (CAY11944); Brg1 ΔNC PrLD (CAY11945); Brg1 YF-to-S PrLD (CAY12228); Brg1 ΔpolyQ PrLD (CAY12222); and Brg1 polyQG (CAY12225). Junction checks were performed with oligos 6429/4438 and 4439/6430.

The WT Bcr1 complementation plasmid, all Bcr1 PrLD deletion plasmids, and all Bcr1 PrLD amino acid mutant plasmids were digested with KpnI/SacI and integrated into the endogenous *BCR1* locus. Plasmids were integrated into a *bcr1Δ/Δ* strain (CAY3008) to yield strains: Bcr1 WT (CAY12231); Bcr1 ΔN PrLD (CAY12234); Bcr1 ΔC PrLD (CAY12237); Bcr1 ΔNC PrLD (CAY12240); Bcr1 YF-to-S PrLD (CAY12394); Bcr1 ΔpolyQ PrLD (CAY12398); and Bcr1 polyQG (CAY12402). Junction checks were performed with oligos 6431/6092 and 4439/6432.

The WT Flo8 plasmid, the Flo8 PrLD deletion plasmid, and all Flo8 PrLD amino acid mutant plasmids were digested with ApaI/SacI and integrated into the endogenous *FLO8* locus. Plasmids were integrated into a *flo8Δ/Δ* strain (CAY9742) to yield strains: Flo8 WT add back (CAY12701); Flo8 ΔN PrLD (CAY12462); Flo8 YF-to-S PrLD (CAY12697); Flo8 ΔpolyQ PrLD (CAY12699); Flo8 polyQG (CAY12700); and Flo8-Taf15 (CAY14266). Correct integration was checked by PCR with oligos 6425/4438 and 4439/6426.

For mNeonGreen-tagged strains, mNeonGreen with was amplified from pRB895 with oligos for the respective TFs to generate homology arms for integration at the C-terminus of the ORF: Efg1, 4446/3685 (8314/3685 for Efg1 ΔC); Brg1, 8312/8313 (8315/8313 for Brg1 ΔC); Flo8, 4994/4995; Bcr1, 8310/8311. Correct integration of constructs was PCR checked for Efg1 with oligos 4478/4438, for Flo8 with oligos 5032/4438, for Brg1 with oligos 8339/4438 and for Bcr1 with oligos 6854/4438.

Prior to use in biofilm and filamentation assays, the *SAT1* cassette was excised from all strains using FLP-mediated excision as previously described [80].

### Biofilm assays

Dry weight measurements of *C*. *albicans* biofilms were carried out as previously described [46]. Briefly, pre-weighed, sterile silicone squares (Bentec) measuring 1.5 cm x 1.5 cm were incubated overnight at 37°C with fetal bovine serum in 12-well plastic plates with shaking. Treated squares were washed with PBS and placed in new 12-well dishes with 2 ml Spider medium per well. *C*. *albicans* strains were grown overnight in YPD medium and 2 x 10^7^ cells added to each well. Plates were then incubated at 37°C with shaking for 90 min for cells to adhere to the silicone squares. Squares were washed with PBS, put in fresh plates with new Spider medium, and incubation continued for 48 hours at 37°C with shaking. Silicone squares were removed, dried overnight, and weighed. The original mass of each square was subtracted from the final mass to determine biofilm dry weight. The control *C*. *albicans* strains used in these assays refer to the TF deletion parental strains with no plasmids integrated (strain CAY3010 for Efg1, Brg1, and Bcr1; CAY9746 for Flo8).

### Filamentation assays

*C*. *albicans* strains were grown overnight in YPD medium at 30°C. Overnight cultures were then diluted 1:30 in fresh Spider medium and grown for 6 hours at 37°C to induce hyphal cell formation. Cells were collected, resuspended in PBS, and imaged immediately. Images were acquired with a Zeiss Axio Observer Z1 inverted microscope using DIC imaging at 40x magnification. The microscope was equipped with AxioVision software (v.4.8) and Zen software (v.3.0 blue edition). At least 10 images were taken per yeast strain tested in different fields of view, and the experiment was repeated at least twice per strain. Post-imaging processing and cell counting was carried out in FIJI (ImageJ v.1.52p) from 5 separate images per strain per experiment (for a total of 200 cells) and classified as yeast, pseudohyphae, or hyphae.

### Yeast cell imaging and quantification of fluorescence

*C*. *albicans* mNeonGreen-tagged mutant strains were grown overnight in SCD (synthetic complete dextrose) medium at 22°C. Overnight cultures were made into wet mount slides and imaged on a Zeiss Axio Observer Z1 inverted fluorescence microscope for fluorescence and DIC imaging at ×63 magnification together with a 1.6X OptoVar. The microscope was equipped with AxioVision software (v.4.8) and Zen software (v.3.0 blue edition). Post-imaging processing was carried out in FIJI (ImageJ v.1.52p).

To quantify TF expression, a perimeter was drawn around the fluorescently labeled nucleus in FIJI and analyzed for mean fluorescence intensity. Background fluorescence was corrected by drawing a perimeter of the same size in a portion of the cell’s cytoplasm and subtracting from the nuclear fluorescence intensity.

### Western blotting

mNeonGreen-tagged TF mutants were grown overnight at 30°C in YPD, diluted to 0.25 OD_600_ in 3 mL YPD, and incubated at 30°C for 5 h to reach mid-log phase. Protein lysates were isolated from mid-log phase cultures via bead beating in RIPA buffer with protease inhibitors (Thermo Fisher) and quantified with a DC Protein Assay (BioRad). 50 μg of protein was loaded onto 4–15% 10-well Mini-PROTEAN TGX stain-free gels (BioRad), one as a protein loading control and one for blotting following transfer to PVDF (Millipore). Primary antibody anti-mNeonGreen (32F6, ChromoTek, 1:1000) was used for mNeonGreen detection. Secondary antibody goat anti-mouse IgG (H+L)-HRP conjugate (Jackson Immunoresearch, 1:10000) was followed by enhanced pico PLUS chemiluminescence (Thermo Fisher). Results were confirmed by two independent experiments. Quantification of western blots was performed using FIJI (ImageJ v.1.52p).

### RNA sequencing analysis

*C*. *albicans* strains were grown overnight in YPD medium at 30°C. Overnight cultures were diluted 1:30 in fresh Spider medium and grown for 6 h at 37°C. RNA was purified from cells with the MasterPure Yeast RNA Purification Kit (LGC Biosearch Technologies) with DNAse I treatment. PolyA RNA was isolated with the NEBNext Ultra II Directional RNA Library Prep Kit for Illumina with NEBNext Poly(A) mRNA Magnetic Isolation Module (NEB) and used to create multiplexed libraries with NEBNext Multiplex Oligos for Illumina (Index Primers Set 1) (NEB). Samples were pooled and sequenced on a NovaSeq 6000.

To measure gene expression, reads were trimmed and aligned to the *C*. *albicans* SC5314 A21 reference genome and assigned to individual A21 genome features using STAR (version 2.7.11a)[85]. Counts were quantified with HTSeq 2.0 (version 2.0.4)[86] as transcripts per million. Differentially expressed genes were identified using DESeq2, with padj < 0.05 and log_2_ foldchange > 2 (Bioconductor version 3.17)[87]. Raw and processed sequencing data were submitted to the NCBI Gene Expression Omnibus (GEO; https://www.ncbi.nlm.nih.gov/geo/) database, accession number GSE245897.

### Mammalian cell culture, live cell imaging, and LacO array analysis

The human U2OS LacO cell line was a gift from the Tjian Lab, and has been previously described [35,47]. Cells were cultured in low-glucose Dulbecco’s modified Eagle’s medium (DMEM, Thermo Fisher Scientific) supplemented with 10% fetal bovine serum (Thermo Fisher Scientific) and 1% penicillin–streptomycin (Thermo Fisher Scientific), and maintained at 37°C with 5% CO_2_. Cells were seeded into 24-well plates with glass bottoms (Cellvis) for live cell imaging experiments. The appropriate plasmid constructs were transfected into each well using Lipofectamine 3000 (Thermo Fisher Scientific) according to manufacturer’s instructions. Cells were grown overnight following transfection, the medium changed to fresh DMEM, and cells imaged with a Zeiss Axio Observer Z1 inverted fluorescence microscope for fluorescence (EYFP and mCherry) and DIC imaging at ×40 magnification. The microscope was equipped with AxioVision software (v.4.8) and Zen software (v.3.0 blue edition). Post-imaging processing was carried out in FIJI (ImageJ v.1.52p).

In order to quantify the LacO array spot bound by different biofilm PrLD LacI-EYFP constructs, a perimeter was drawn around the array in FIJI and the spot analyzed for both fluorescence intensity and area. Background fluorescence intensity was corrected for by subtracting the fluorescence signal immediately outside the array spot in cell nuclei. Quantification of mCherry signal at the LacO array bound by PrLD-LacI-EYFP constructs was determined as previously described [35]. Briefly, the array spot was located in the EYFP channel and then the mCherry signal measured for maximum fluorescence intensity at the array (I_peak_). Two locations adjacent to the array within a ~2 μm radius were measured in the mCherry channel and their fluorescence intensities averaged (I_periphery_) to represent background fluorescence in cell nuclei. The mCherry-PrLD enrichment at the LacO array was calculated as the ratio of the peak signal divided by the background signal (I_peak_/ I_periphery_).

### Hexanediol treatment of U2OS nuclear condensates

U2OS LacO cells transfected with PrLD LacI-EYFP constructs were treated with 1,6-hexanediol (Sigma-Aldrich) or 2,5-hexanediol (Thermo Fisher Scientific). Compounds were prepared in pre-warmed DMEM at 20% mass:volume concentrations. Media was removed from U2OS LacO cells growing in 24-well glass bottom dishes and replaced with 1 ml fresh DMEM so that addition of 1 ml hexanediol medium yielded final concentrations of 10% 1,6- or 2,5-hexanediol. Cells were imaged directly before hexanediol treatment and at 5 min post-treatment, with additional images acquired every 10 sec with a Zeiss Axio Observer Z1 microscope for fluorescence (EYFP) and DIC imaging at ×40 magnification. Time point t = 0 s corresponds to cells directly before hexanediol addition, and t = 300 s corresponds to cells after 5 min of hexanediol treatment. Condensates not associated with the array spot were quantified via counting in FIJI (ImageJ v.1.52p).

### Protein purification

Protein constructs in a 6xHis-MBP-TEV protease site expression vector were transformed into BL21 (DE3) Star *E*. *coli* cells. Cells were grown overnight at 37°C in Luria broth (LB) medium, diluted 1:100 in fresh LB the following day, grown at 37°C to an OD_600_ of 0.5–0.7, and induced with 1 mM isopropyl β-D-1-thiogalactopyranoside. MBP-Efg1 was induced at 25°C overnight. MBP-Flo8, MBP-GFP-CTD (26 heptad repeats plus the 12-residue C-terminus sequence of *C*. *albicans* Rpo21 CTD, ORF bases 4605–5187), and MBP-GFP were induced at 30°C for 4 hours. Cells were then lysed with lysozyme and sonicated in lysis buffer made up of 10 mM Tris, pH 7.4, 1 M NaCl, 1 mM phenylmethylsulfonyl fluoride and a protease inhibitor cocktail (Thermo Scientific Pierce Protease Inhibitor). The resulting protein was purified by nickel affinity column chromatography, followed by size exclusion column chromatography on a Sephacryl S300 26/60 column (GE Healthcare). Protein fractions were collected and concentrated in Amicon Ultra 50K concentrators (Millipore), then frozen in liquid nitrogen and stored at -80°C until use in phase separation assays.

### Phase separation assays

Proteins were thawed at 22°C and diluted into 10 mM Tris-HCl, pH 7.4, 150 mM NaCl buffer. Proteins were concentrated in Amicon Ultra 0.5-ml centrifugal filter units (Millipore) to a volume of 100 μl and concentrations determined using a Nanodrop 2000c (Thermo Fisher Scientific). Proteins were further diluted in 10 mM Tris-HCl buffer with 150 mM NaCl to appropriate concentrations for each assay. Reactions with TEV were set up in 10 μl total volumes (9.5 μl protein with 0.5 μl of 0.3 mg/ml of TEV) and incubated for 30 min at 22°C. Where noted, 5% PEG-8000 was included as a molecular crowding agent. For gDNA phase separation assays, SC5314 gDNA was diluted in the same buffer as the proteins and added at a concentration of 50 nM before TEV treatment. Following incubation, proteins were immediately imaged in 10-well chamber slides (Polysciences) with 2.5 μl protein solution per well sealed under a glass coverslip. Images were acquired with a Zeiss Axio Observer Z1 inverted fluorescence microscope for fluorescence and DIC imaging at 63x magnification. Post-imaging processing was carried out in FIJI (ImageJ v.1.52p).

### Partitioning of RNA Pol II GFP-CTD into Efg1 and Flo8 droplets

The GFP-CTD fusion protein was concentrated in 10 mM Tris-HCl, pH 7.4, 150 mM NaCl buffer and then diluted 10 mM Tris-HCl buffer with 150 mM NaCl and 5% PEG-8000 to 15 μM. Efg1 and Flo8 were concentrated and diluted as described above with the addition of 5% PEG-8000 to the protein buffer. Efg1 and Flo8 were present at 15 μM and the GFP-CTD protein (or GFP alone as a control) added at a 1:10 dilution for a final concentration of 1.5 μM. Proteins were incubated at 22°C for 30 min in 10 μl volumes with TEV and imaged in chamber slides. Images were acquired with a Zeiss Axio Observer Z1 inverted fluorescence microscope. FIJI (ImageJ v.1.52p) was used to calculate fluorescent signals. To calculate GFP-enrichment ratios, mean fluorescence intensity signal per unit area inside each Efg1 or Flo8 condensate was divided by the mean fluorescence intensity signal outside the condensates, after subtracting background fluorescence signal. The background signal was calculated for images of either Efg1 or Flo8 condensates without the presence of GFP-CTD.

### Mouse infections and imaging of tissue sections

To analyze GI colonization, strains CAY12633/CAY14163 were used to evaluate *EFG1* (WT and YF-to-S mutant) and strains CAY11942/CAY12228 were used to evaluate *BRG1* (WT and YF-to-S mutant). We note that *EFG1* strains had two copies added back to an *efg1Δ/Δ* mutant as strains carrying one copy of *EFG1* can rapidly lose that copy by recombination [81].

Yeast strains were grown overnight at 30°C in 3 ml of YPD medium. The following day, 500 μl of overnight culture was added to 9.5 ml of YPD and grown for 4 hours. Cells were pelleted, washed with H_2_O, and resuspended in 1 ml H_2_O. Cells were counted by hemocytometer and an inoculum of 2 x 10^8^ cells/ml was made for each strain. BALB/c mice (2 mice per strain) were inoculated with 500 μl of the prepared yeast dilutions. Mice were given a standard chow diet and antibiotic-treated water (2 mg/mL streptomycin, 1.5 mg/mL penicillin, 2.5% glucose). Yeast strains were allowed to colonize for 7 days after which time the colons were processed for imaging.

Tissue sections were immersed in methacarn for 24 hours, washed twice with 70% ethanol and embedded in paraffin blocks. Tissue sections were rehydrated in xylene and subsequent ethanol washes, followed by incubation in blocking buffer (1% horse serum in PBS) for 30 min at room temperature. A 1:500 dilution of anti-*Candida albicans* antibody (Fitzgerald Industries International) was added to tissue sections and incubated overnight at 4°C. Tissue sections were washed and stained at 22°C for 1 hour with a 1:250 dilution of WGA1 and UEA1 coupled to Rhodamine (Vector Laboratories) in PBS, and a 1:500 dilution of DAPI (Thermo Fisher Scientific) in PBS. Following incubation, tissues were rinsed with PBS and mounted on slides with anti-fade mounting media (Thermo Fisher Scientific). Slides were imaged at 40x magnification with a Zeiss Axio Observer Z1 inverted fluorescence microscope. Cell counts were performed for each yeast strain in at least 3 tissue sections per mouse, with at least 300 cells counted per strain. Post-image processing was performed in FIJI (ImageJ v.1.52p).

## Supporting information

S1 Fig Seven of nine biofilm network TFs contain PrLDs. Each biofilm TF was analyzed with the PLAAC algorithm to identify PrLDs.
(TIF)

S2 Fig TF PrLD mutants display similar nuclear localization and intensity. **(A)** WT and mutant TFs were C-terminally-tagged with mNeonGreen and visualized by microscopy. Scale bar; 10 μm. **(B)** Mean fluorescence intensity was quantified with FIJI by subtracting the intensity of a 5 pixel diameter circle outside the nucleus from the intensity of a 5 pixel diameter circle inside the nucleus. 15 different cells were quantified for each strain. All statistical tests were performed using ordinary one-way ANOVA with Dunnett’s multiple comparisons test, in which the mean value for each mutant strain was compared to the mean value for the control. Error bars show S.E.M. *P < 0.05; ****P < 0.0001; ns = not significant.
(TIF)

S3 Fig Analysis of Bcr1 TF PrLD mutant expression in *C*. *albicans*. **(A)** Representative western blot of Bcr1 PrLD mutants. Overnight cultures of mutant strains were grown to mid-log phase at 30°C for 5 h in YPD. Protein lysates were analyzed by western blotting with anti-mNeonGreen antibody to detect Bcr1-mNeonGreen expression. **(B)** Representative protein loading control of Bcr1 PrLD mutants, used to correct for expression in Bcr1 quantification. **(C)** Quantification of Bcr1 PrLD mutants, displayed as a ratio of Bcr1 mutant expression over protein loading controls.
(TIF)

S1 Appendix Differentially expressed genes with > 4-fold change between WT and YF-to-S mutants for Efg1 and Brg1. (XLSX)

S2 Appendix Normalized gene counts for WT and YF-to-S mutants of Efg1 and Brg1. (XLSX)

S3 Appendix Plasmids, oligonucleotides, and strains used in this study. (XLSX)
