# Supplementary figures and images for: Filamentation and biofilm formation are regulated by the phase-separation capacity of network transcription factors in Candida albicans
Source: PLoS Pathog. 2023 Dec 13;19(12):e1011833. doi: 10.1371/journal.ppat.1011833 (PMC10718430; doi:10.1371/journal.ppat.1011833)

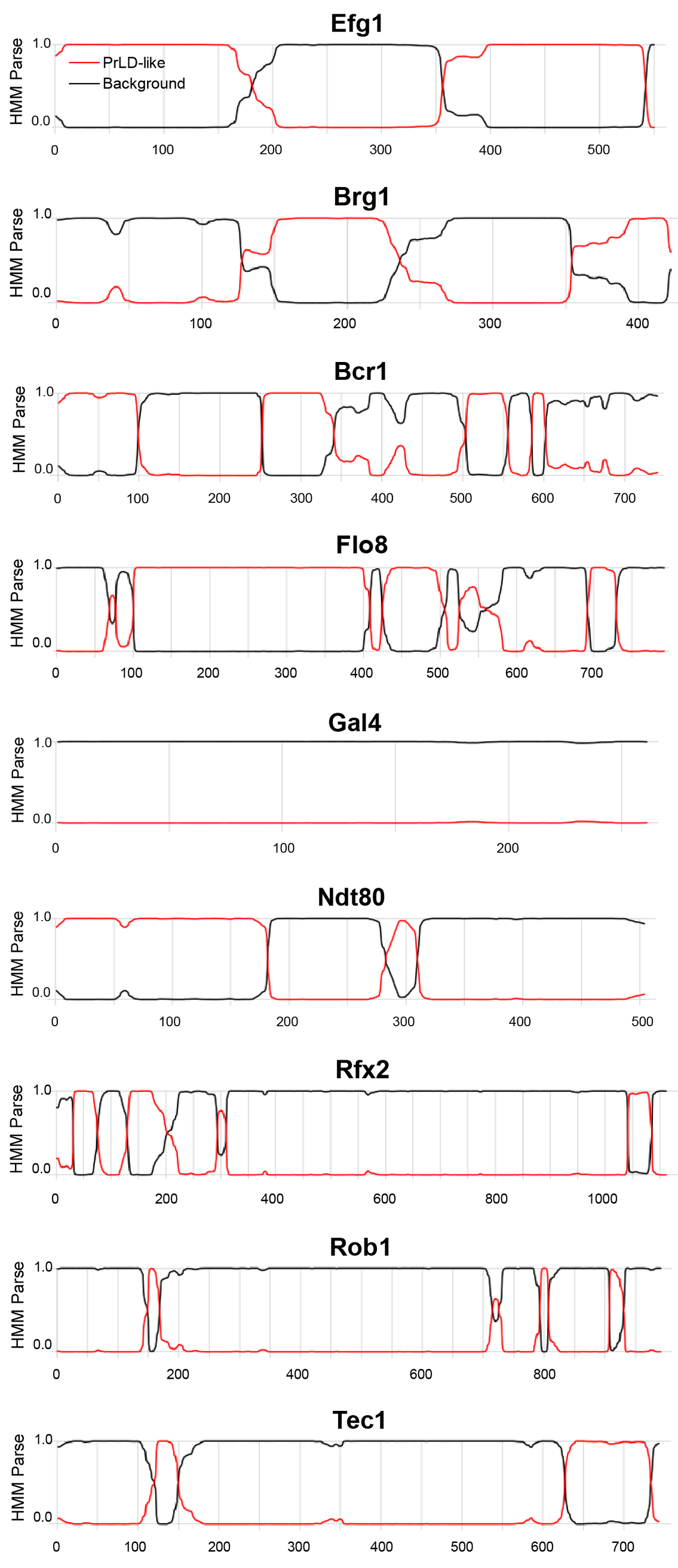

Supplement: S1 Fig — Each biofilm TF was analyzed with the PLAAC algorithm to identify PrLDs. (TIF) [file ppat.1011833.s001.tif]

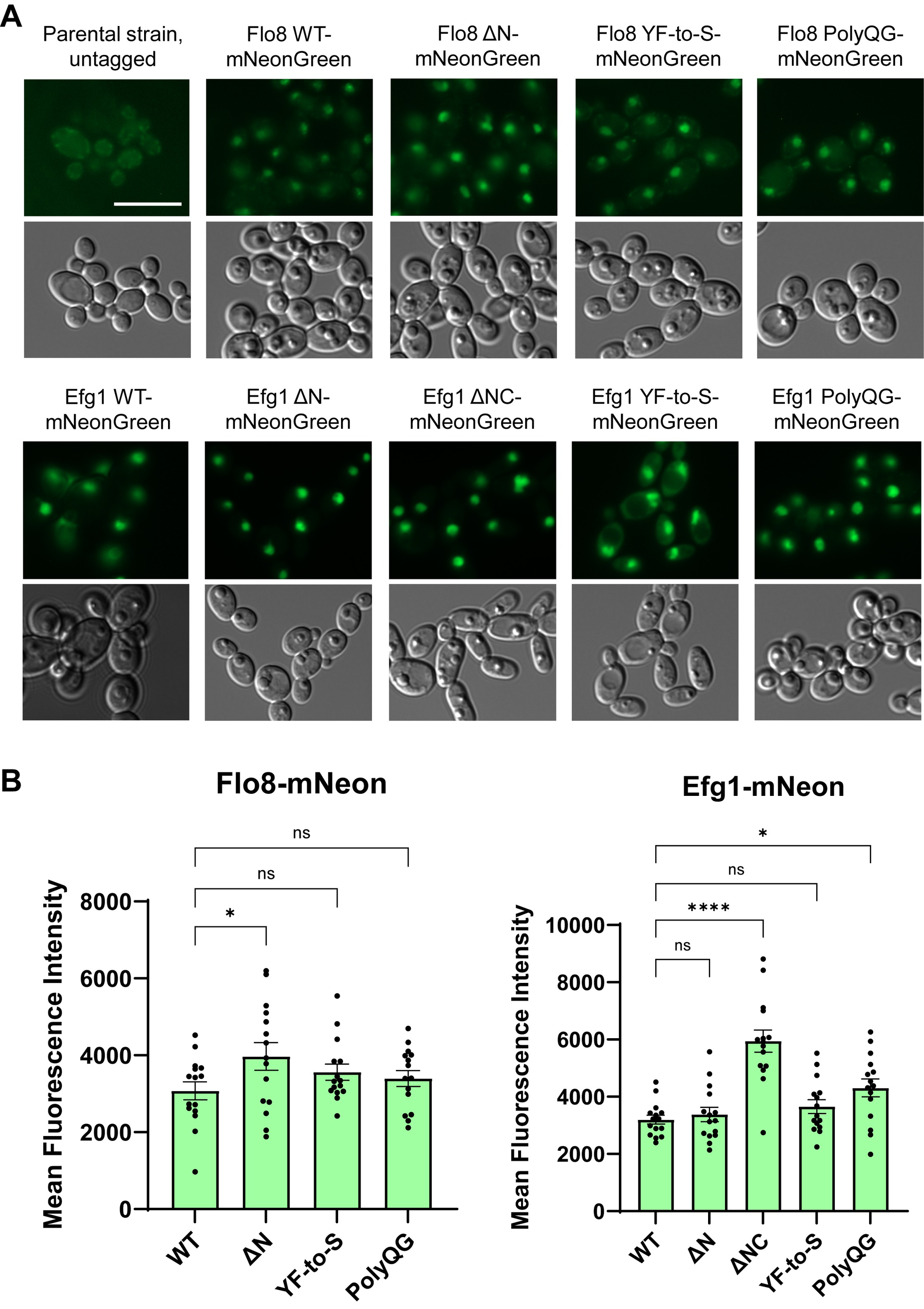

Supplement: S2 Fig — (A) WT and mutant TFs were C-terminally-tagged with mNeonGreen and visualized by microscopy. Scale bar; 10 μm. (B) Mean fluorescence intensity was quantified with FIJI by subtracting the intensity of a 5 pixel diameter circle outside the nucleus from the intensity of a 5 pixel diameter circle inside the nucleus. 15 different cells were quantified for each strain. All statistical tests were performed using ordinary one-way ANOVA with Dunnett’s multiple comparisons test, in which the mean value for each mutant strain was compared to the mean value for the control. Error bars show S.E.M. *P < 0.05; ****P < 0.0001; ns = not significant. (TIF) [file ppat.1011833.s002.tif]

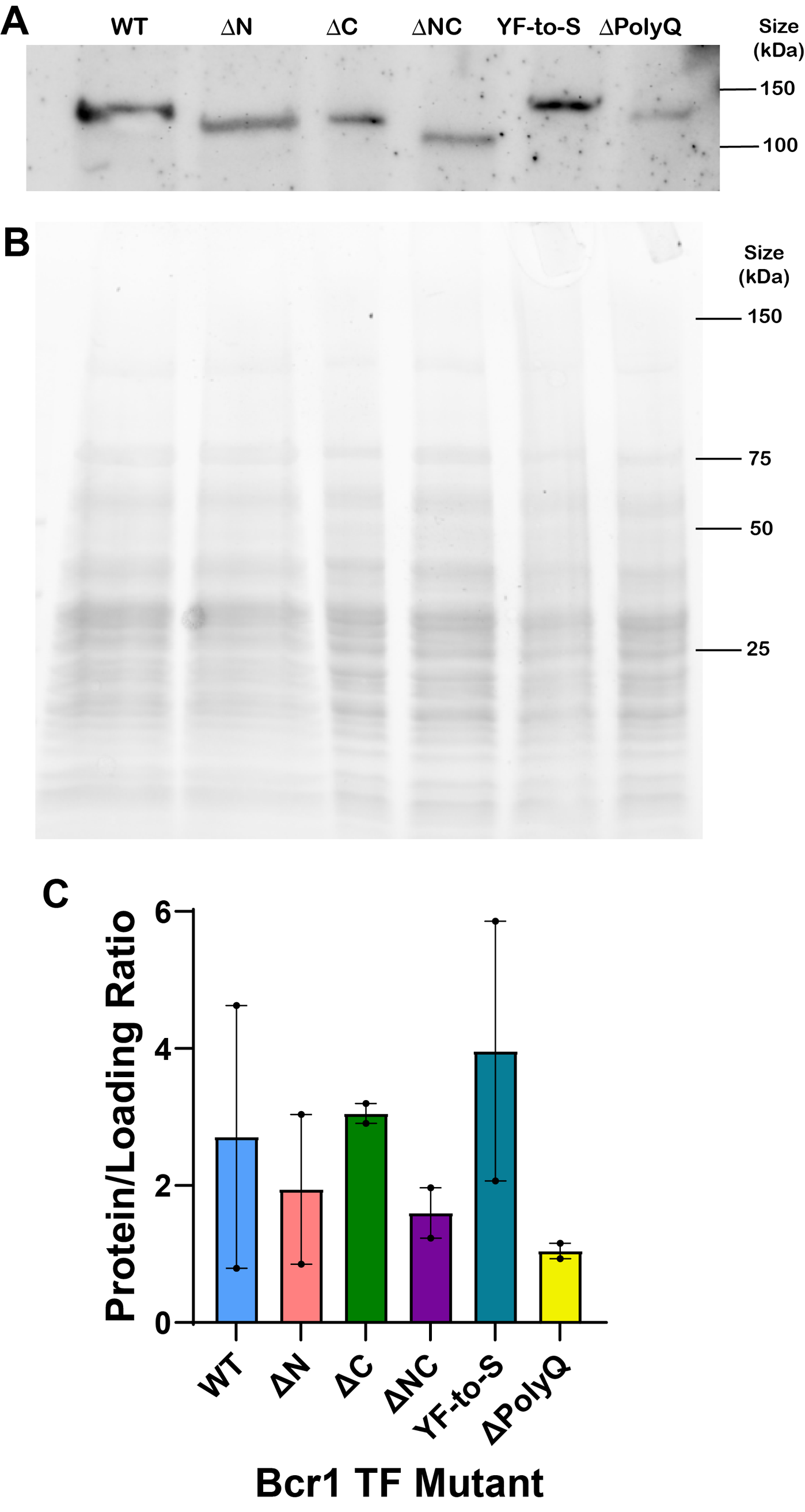

Supplement: S3 Fig — (A) Representative western blot of Bcr1 PrLD mutants. Overnight cultures of mutant strains were grown to mid-log phase at 30°C for 5 h in YPD. Protein lysates were analyzed by western blotting with anti-mNeonGreen antibody to detect Bcr1-mNeonGreen expression. (B) Representative protein loading control of Bcr1 PrLD mutants, used to correct for expression in Bcr1 quantification. (C) Quantification of Bcr1 PrLD mutants, displayed as a ratio of Bcr1 mutant expression over protein loading controls. (TIF) [file ppat.1011833.s003.tif]
